# The global structure of classroom social networks predicts academic and psychosocial adjustment

**DOI:** 10.1093/pnasnexus/pgag291

**Published:** 2026-08-26

**Authors:** Claudia Neuendorf, Naoki Masuda, Kou Murayama

**Affiliations:** Hector Research Institute of Education Sciences and Psychology, University of Tübingen, 72074 Tübingen, Germany; Department of Education Science, University of Potsdam, 14476 Potsdam, Germany; Gilbert S. Omenn Department of Computational Medicine and Bioinformatics, University of Michigan, Ann Arbor, MI 48109, USA; Department of Mathematics, University of Michigan, Ann Arbor, MI 48109, USA; Department of Mathematics, State University of New York at Buffalo, Buffalo, NY 14260, USA; Institute for Artificial Intelligence and Data Science, State University of New York at Buffalo, Buffalo, NY 14260, USA; Center for Computational Social Science, Kobe University, Kobe, 657-8501 Hyogo, Japan; Hector Research Institute of Education Sciences and Psychology, University of Tübingen, 72074 Tübingen, Germany; Research Institute, Kochi University of Technology, 782-8502 Kami, Japan

**Keywords:** social networks, machine learning, education

## Abstract

Humans are deeply social, and in early life, classroom friendships shape academic and socio-emotional development. Classroom peer ecologies describe the social structure in which individual relationships are situated and which form the context for student learning and development within these classrooms. Yet, research has only begun to explore how these global social network structures are related to classroom-level outcomes. Using a large-scale dataset of 35,038 students in 1,433 classrooms, we extracted 314 social network metrics from four different network types (i.e. friendship, rejection, help, and break-time contacts) and applied machine learning to predict various classroom-level outcomes, including academic achievement, motivation, and social integration. Classroom networks predicted both academic achievement (up to 2% of variance) and well-being (up to 18% of variance), beyond school tracks, socioeconomic status, and demographic factors. However, they did not significantly improve predictions for other characteristics of the teaching and learning environment—such as instructional methods and motivational characteristics. The predictive power for different outcomes was highly dependent upon the type of network and categories of network metrics. These insights position social networks as a powerful tool for understanding aspects of educational environments and for informing network-based interventions.

Significance Statement Classroom friendships and peer interactions shape how children learn and feel at school, yet little is known about how whole-class social structures influence educational outcomes. Analyzing 35,038 students across 1,433 classrooms, we find that global properties of classroom social networks—spanning friendships, rejection, helping, and everyday interactions—contribute to academic performance and social integration, beyond demographic and structural features of schools. Our results suggest that different social dynamics relate uniquely to educational and psychosocial adjustment. By revealing how classroom social structures are related to academic and socio-emotional functioning, this work points to the potential of network-informed approaches for understanding learning environments and designing interventions that promote both achievement and well-being.

## Introduction

The classroom is one of the most important contexts setting the stage for human development (1, 2). Children and adolescents spend much of their daily lives in classrooms where they encounter many social situations through which they build and refine the psychosocial skills essential for their later adjustment. For example, they form and maintain friendships through cooperation and reciprocity, negotiate and compete with equal-status others, deal with disagreements and conflict, forge alliances, and construct a social system governed by their own rules and norms (2, 3). These relationships are in stark contrast to the ones they have with adults—such as parents or teachers—where there is a clear power difference, and the roles are largely fixed (3–5). In fact, a broad array of research has shown that individual social experiences in classrooms, such as having close friends, being accepted by others, receiving social support, or being rejected or excluded by peers, significantly influence young people's academic (6), motivational (7), physical (8), and psycho-emotional (9) flourishing.

While these individual social experiences are undeniably important, there has been a notable gap in research examining the role of “classroom-level” group dynamics in education systems (10). The classroom social structure or “classroom peer ecology” as a whole shapes opportunities for learning, belonging, and behavior in ways that cannot be reduced to individual-level interactions. Social-cognitive theories (3, 11), for example, highlight that contact and interactions between diverse peers foster cognitive development and learning, implying that peer ecologies characterized by certain structural features (e.g. low homophily) may support achievement and motivation. Social learning theory (12) further suggests that classroom hierarchies can influence whose behavior becomes normative. Highly popular, high-achieving peers may set group norms that shape the learning environment (13) to become more academically oriented (14), while the popularity of aggressive children will undermine the overall classroom disciplinary climate (e.g. 15). The literature on teachers’ “invisible hand” in peer ecology (16) additionally emphasizes that teacher practices like group composition, seating arrangements, or their attunement to the peer ecology and students’ social and emotional learning can shape relationship patterns. Together, these perspectives indicate that peer ecologies may relate to a wide range of outcomes at the individual level (achievement, motivation, and socio-emotional functioning), the classroom level (climate, behavioral norms, and shared perceptions of learning), and the teacher level (instructional practices and attunement).

Peer ecologies operate through the complex global structure of relationships in a classroom instead of isolated dyadic experiences. Thus, they are captured through classroom-level metrics of sociometric networks that describe the pattern of relationships between all students in a classroom (2). Despite its clear theoretical importance, empirical research on the effects of the peer ecology itself has been significantly limited not only in number but also in its scale, with most studies relying on small samples (typically 20–60 classrooms) and focusing on a narrow and different set of outcome variables and network features. A key bottleneck driving this situation is the myriad ways in which peer ecologies can be quantified. Classroom social networks can be characterized by numerous features capturing information regarding hierarchy, cohesion, subgroup formation, or prevailing popularity norms, among others. These features are often highly interrelated, and theory provides little guidance on which specific feature best represents a construct of interest. Thus, studies often arbitrarily select a narrow range of network features, and it remains unclear whether these are in fact the most informative ones. As a result, past empirical research remains fragmented and has not come close to fully exploiting the potential influence of classroom social network structures. This situation not only constrains cumulative knowledge but also risks overlooking patterns that do not neatly follow existing theoretical expectations (17).

Here, we address this fundamental research gap by leveraging machine-learning approaches to systematically explore complex, interrelated indicators, and evaluate their predictive value across multiple outcomes. Our proposed approach can (i) incorporate a broad set of theoretically motivated and data-driven network indicators without requiring strong a priori assumptions about their relative importance; (ii) leverage machine-learning methods that are explicitly designed to handle multicollinearity and high-dimensional predictor spaces; (iii) facilitate the analysis of multiple student, classroom, and teacher outcomes while guarding against inflating type I error through out-of-sample validation; and (iv) support discovery and theory development by revealing which aspects of classroom social networks consistently show predictive value.

Specifically, we analyzed the largest possible social network dataset of classrooms collected in Germany, using data from 1,433 classrooms from ninth-grade school children (average classroom size = 24). These recently released data from a government-commissioned national assessment offer exceptionally high-quality data, far surpassing the quality that is typically available in similar studies. For each classroom, students rated their relationships with every other student in the same class with regard to four different types of relationships (i.e. friendship, helping behavior, spending break time together, and rejection), thus allowing us to calculate a comprehensive set of more than 300 different classroom-level social network indicators. We integrated social network analysis and machine learning to explore this unprecedentedly large dataset in education science. Specifically, we assessed the extents to which these network features could jointly predict a variety of 33 academic outcomes at the classroom level, including psychosocial, motivational, and cognitive outcomes, as well as the academic climate of the classroom. Moreover, we determined the relationships between social network features and instructional characteristics, as instruction represents the way in which teachers shape and respond to interactions in the classroom. This “all-inclusive approach” (18) provides a holistic picture of the role of the classroom peer ecology. It further identifies outcome domains that are more closely linked to the peer ecology and are most promising targets for further research and theory development.

## Methods

### Data

We used data from the IQB-Trends in Student Achievement Study in 2018 (19, 20). This is a German national large-scale assessment of student competences and is conducted in regular cycles. In 2018, the focus was on testing students’ mathematics and science competence. The study sample was representative of the German population of ninth graders and was conducted in all school types in the German tracking system. Apart from students, data were collected from the students’ parents as well as from the teachers and principals of the participating schools. For the current paper, only data from students and teachers were used. For classrooms in which multiple teachers responded to the questionnaires, we averaged the teachers’ response values. Because the assessment is part of the national educational monitoring, participation in the competence assessment was compulsory for all students. Participation in the student and teacher questionnaires was compulsory in only some of the federal states. Written parental consent was obtained in cases where students’ participation was voluntary. Teacher data were collected using an online questionnaire. Teachers were informed of the study's data protection provisions. All participants could decline to answer any of the questions. All questionnaires, informed consent forms, and the procedure were reviewed and approved for compliance with data protection regulations and ethical standards by the data protection officers and the ministries of education of the 16 German federal states.

#### Sample

In 2018, a total of *n* = 44,941 ninth-grade students (mean age *M* = 15.6) from *n* = 1,473 schools (*n* = 2,292 classrooms) participated in the study. In schools of the highest track, one classroom was randomly selected for participation, while in other tracks, two classes were selected. In our analyses, we included 1,433 classes for which social network ratings (see below for details) were available from at least three students, at least 70% of the students in a classroom participated, and all chosen network features were computable.^a^ The average classroom size was 24.5 students (SD = 4.2). Table A1 in the supplement presents details about the student sample by academic track. Table A2 shows classroom-level descriptives.

### Outcome measures

This research used a broad array of outcome variables to examine the extent to which classroom social network structures could predict these outcome variables. These variables were constructed from student and teacher questionnaires and fell into different categories.

#### Cognitive outcomes

We considered the classroom mean and dispersion of student achievement tests. Because the 2018 cycle of the Trends in Student Achievement Study focused on mathematics and science, we could use achievement scores in mathematics, biology, chemistry, and physics. The tests were constructed to have curricular validity (19). They were administered with a multiple-matrix booklet design and scaled using Item Response Theory (21). We used Warm's weighted likelihood estimates (22) as estimates of achievement. Intra-class correlation coefficients (23) showed that between 35 and 53% of the variance could be attributed to the class level (ICC), and the group-size-adjusted group-level reliability *λ* (also known as ICC(2)) was between 0.92 and 0.96 for the four achievement tests.

#### Socio-emotional outcomes

Socio-emotional outcomes included student responses to the scales “social integration in the classroom” and “well-being in school.” Social integration comprised seven items that tapped into the feeling of being socially accepted and having friends in the classroom (24). Reliability was moderate to high (Cronbach's *α* = 0.82, *λ* = 0.65), and a considerable amount of variation existed within rather than between classrooms (ICC = 0.08). Well-being in school asked not only about students’ feelings that they were socially accepted and integrated in school but also whether they felt lonely and out of place (24). It consisted of nine items (e.g. “I feel lonely in school”; Cronbach's *α* = 0.85, *λ* = 0.65, ICC = 0.08). We also included a teacher variable comprising items about teacher stress (25) (11 items, *α* = 0.77) in this category. Teachers were asked how often different aspects contributed to their feelings of stress. These aspects included, for example, the size of the class, students’ achievement heterogeneity, implementing special instructional formats, and workload. Teachers responded on a 4-point scale ranging from 1 (never) to 4 (often).

#### Motivational outcomes

Motivational outcomes included four scales of “interest” in mathematics, biology, chemistry, and physics. Each of the subjects was assessed with four items asking about personal importance, enjoyment, and interest in the subject (26). Reliability was high (Cronbach's *α* = 0.89) but with considerable variations within classrooms (*λ* between 0.51 and 0.68, ICCs between 0.04 and 0.08). We also included “mathematics anxiety” (scale adapted from Mang et al. (27)) as a fifth motivational outcome variable (Cronbach's *α* = 0.88, *λ* = 0.47, ICC = 0.04). Items referred to worrying about getting poor grades in mathematics, having difficulties on mathematics tests, or being anxious while studying and being nervous when failing to complete a task. For all items, students had to respond on a scale ranging from 1 (does not apply at all) to 4 (completely applies).

#### Instructional methods

**Figure 1:**
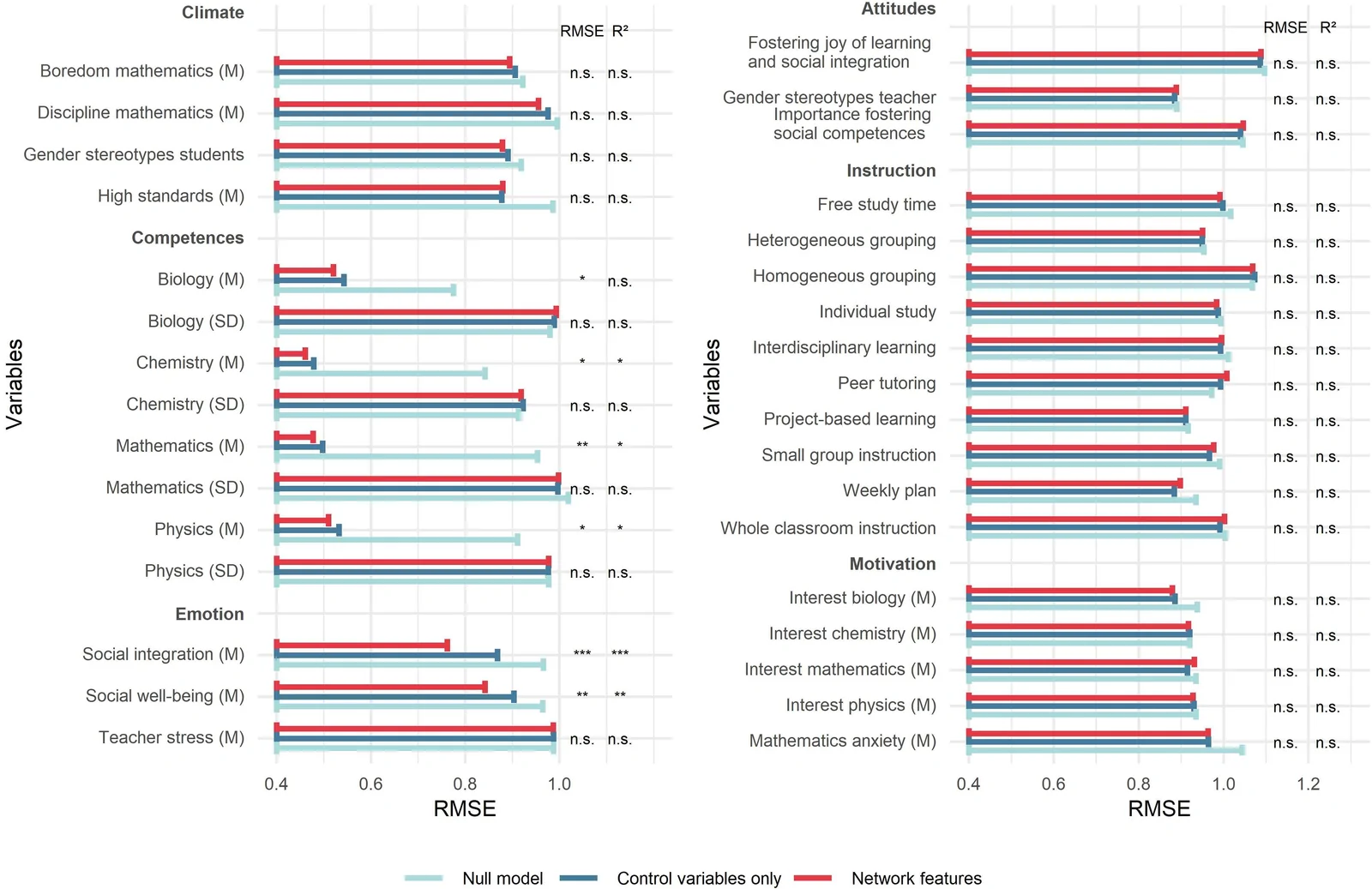
Prediction errors of the elastic net regressions for each outcome variable. The figure shows prediction errors (RMSE) across the three models with successively more variables (bottom: null model, baseline prediction of test data by training data mean of the outcome, middle: model with control variables only, top: model including control variables and network variables). Stars denote *P*-values indicating whether there was a significant decrease in the prediction error of the model including network variables beyond the model with control variables only. For exact *P*-values, refer to Table E1 in Supplement E. Results were based on predictions for 372 classrooms in the hold-out data. Significance testing was based on 1,000 bootstrap iterations. All tests were two-sided. *P < 0.05. **P < 0.01. ***P < 0.001.

Teachers were asked which instructional formats they used in the class. For each method, they could indicate how often they used each of them (1 = never, 2 = a couple of times per year, 3 = a couple of times per month, and 4 = a couple of times per week). The individual instructional formats are shown in Fig. 1. The teachers were also asked about their approach to differentiation and were asked to indicate how often they used homogeneous grouping and heterogeneous grouping methods (e.g. “I group students with different ability levels together”) on a scale ranging from 1 (never) to 4 (often).

#### Academic climate

Academic climate included the extent to which students felt bored in mathematics class (28, 29). Boredom was assessed with three indicators, which we averaged for this study (e.g. “In mathematics class, I feel very bored”; *M* = 2.16, SD = 0.27, *λ* = 0.61, ICC = 0.07). Second, we included a scale measuring the disciplinary climate of the mathematics class. Disciplinary climate comprised three items, asking about the extent to which students were disruptive, talking loudly, or horsing around during lessons (30) (“In our mathematics class, students horse around”; Cronbach's *α* = 0.91, *λ* = 0.92, ICC = 0.34). The answer categories ranged from 1 (does not apply) to 4 (completely applies). Third, students’ reports of academic standards in their classroom were assessed with eight items asking how cognitively demanding the tasks assigned by the mathematics teacher were (“Our teacher gives us tasks that force us to think”; Cronbach's *α* = 0.79, *λ* = 0.78, ICC = 0.14, *M* = 2.25, SD = 0.25). Finally, another dimension of academic climate in mathematics we included was the extent to which students agreed with statements presenting gender stereotypes about mathematics achievement and motivation (“Who is better at mathematics?”; 1 = applies to girls, 2 = applies more to girls than boys, 3 = boys/girls the same, 4 = applies more to boys than girls, and 5 = applies to boys; *M* = 3.17, SD = 0.42, *λ* = 0.79, ICC = 0.14).

#### Teachers’ attitudes and values

We included teachers’ responses to questions about their aims in educating students. Specifically, they were asked whether, for them, the main aim of school was the fostering of social competence (*M* = 3.28, SD = 0.38) or the fostering of social integration and the joy of learning (*M* = 3.14, SD = 0.42). All items were answered on a 4-point scale. Finally, gender role stereotypes about abilities and motivation in mathematics for boys and girls were assessed. Teachers responded to five items with an answer format of five categories (1 = applies to girls, 2 = applies more to girls than boys, 3 = boys/girls the same, 4 = applies more to boys than girls, and 5 = applies to boys). The mean across all teachers was *M* = 3.10 (SD = 0.29, Cronbach's *α* = 0.72).

Supplement A2 presents all scales’ descriptive statistics aggregated at the classroom level, which is how they were used in the current study. Table A4 presents the wordings of all individual items. The original German wording can be found in the scale documentation available from the Institute for Educational Quality Improvement (28).

### Network features

The network features were based on the students’ responses to the social network questionnaires. For each student, the questionnaire contained a list of all of that student's classmates so that the student could refer to each classmate. For each classmate, students had to respond to four questions. By checking a box, they had to indicate (i) whether the other student was their friend (“friendship”), (ii) whether they spend time during breaks with the other student (“break time”), (iii) whether they ask the other person for help when they have a problem (“help”), and (iv) whether they would prefer not to sit next to this student (“rejection”). Then, a binary multilayer network was created for each classroom. On average, students nominated *M* = 10.13 (SD = 5.66) individuals as friends, indicated that they spend their time with *M* = 5.02 (SD = 3.6) classmates, that they would ask *M* = 4.65 (SD = 4.34) classmates for help, and that they would reject *M* = 5.07 (SD = 4.90) classmates as a seat neighbor. Classroom-level densities (i.e. the total number of nominations divided by the maximum possible number) for each variable were *M*_friend_ = 0.43, *M*_contact_ = 0.22, *M*_help_ = 0.20, and *M*_reject_ = 0.22.

We included a total of 314 network features. We calculated indicators described in the standard sociological literature on social network analysis (31), indices used by other studies of classroom structure (32–37), and additional indices available in network science (38, 39). Table A3 in Supplement A presents the full list of social network features in our analyses. The network features were mostly computed with the *igraph* package available for R and Python (40). If two features were correlated more than *r* = |0.95|, one of them was dropped from the analysis, as they represent effectively identical inputs with no unique variance (*n* = 2). Supplement B (Excel file) includes a list of all features, their descriptions, their formulas, and their distributions in our sample. The network features included indicators calculated from each of the four networks (*n* = 218; e.g. density, diameter, centralization, clustering coefficient, coreness, connectedness, and clique size), indicators that included network and actor attributes (*n* = 84; e.g. assortativity, Moran's *I*, popularity of students with certain characteristics), and multiplex indicators of the co-occurrence of nominations between the different network questions (*n* = 12; e.g. tie overlap and degree correlation). The four networks each conveyed distinct social information. The average proportion of overlapping ties (i.e. the Jaccard similarity) ranged between *M*_friendship; help_ = 0.34 and *M*_friendship; break time_ = 0.43 among the positive networks, and from *M*_1-rejection; help_ = 0.21 to *M*_1-rejection; friendship_ = 0.44 when using the inverse of the rejection network. These values indicate moderate overlap but substantial uniqueness across networks. Furthermore, pairwise correlations between corresponding network features across the four network types averaged *M*_|*r*|_ = 0.29 (*Q*_0.25_ = 0.10, *Q*_0.75_ = 0.44), suggesting that each network question contributed unique and nonredundant information (see Fig. A1 in Supplement A).

#### Clustering and grouping network features

**Figure 2:**
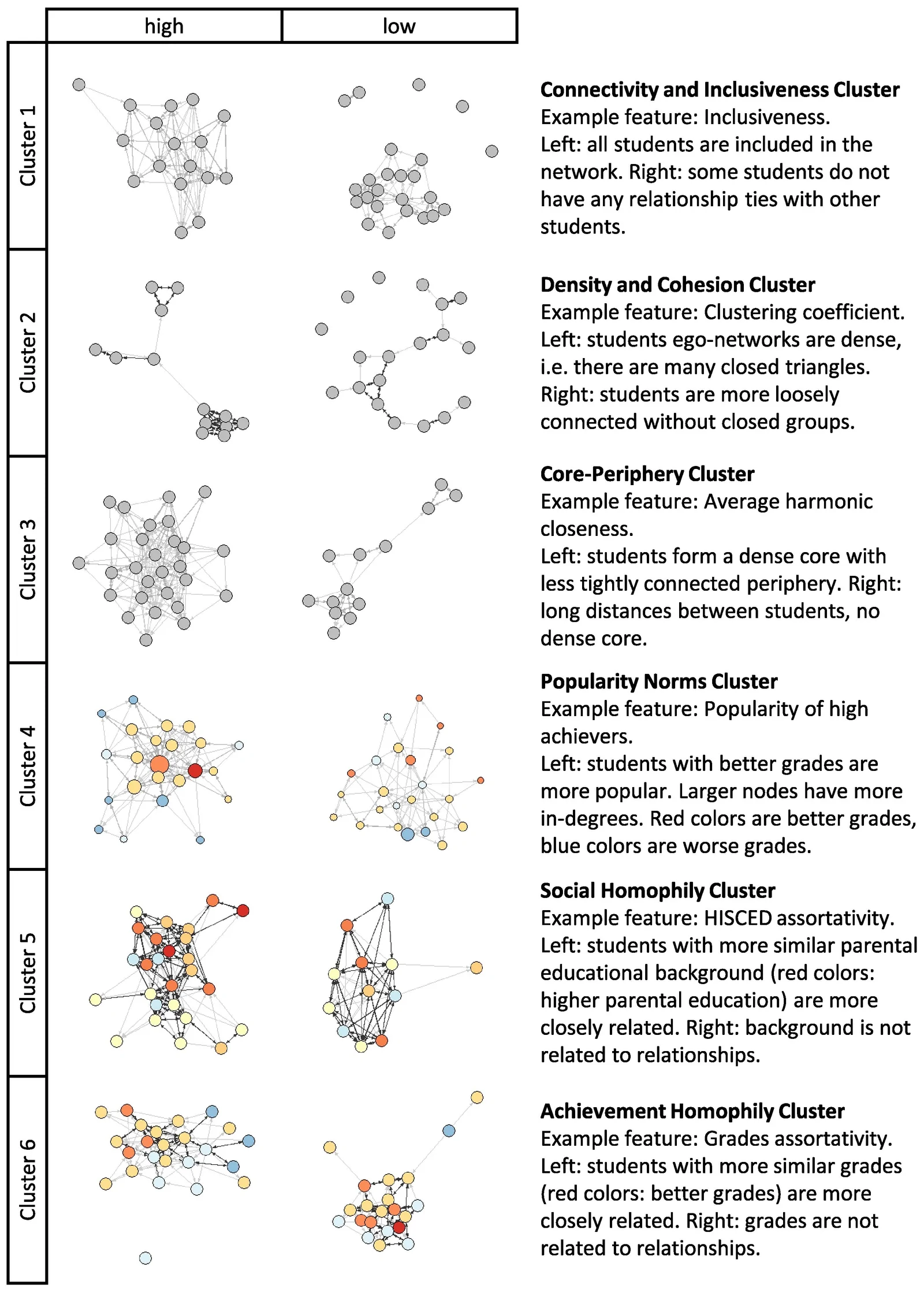
Examples of specific network feature clusters. Each network represents one classroom from the original dataset.

To deal with multicollinearity and to obtain interpretable results, we clustered the network features, first on a theoretical basis, by dividing them into sets based on the type of network (i.e. friendship, break time, help, rejection, and multiplex indicators, which referred to multiple networks at the same time). Second, we applied a k-medoids clustering algorithm to the 314 × 314 correlation matrix between all pairs of network indicators, separately for each of the four networks (friendship, break time, help, and rejection). Details on how the clustering procedure was conducted can be found in Supplement C. The procedure led to six clusters: Cluster 1 (“Connectivity and Inclusiveness Cluster”) comprised *n* = 14 features that were mainly related to connectedness, i.e. the degree to which there are groups or individual actors with no connection to another part of the network (e.g. components and isolates). Cluster 2 (“Density and Cohesion Cluster”; *n* = 26) was the largest cluster and contained features related to both density and distances in the network. Variables in Cluster 3 (“Core-Periphery Cluster”; *n* = 9) featured variables that all had fairly high correlations with network size and were related to a more distinct core-periphery structure, such as closeness, clique size, embeddedness, and coreness (41). Research has shown that larger networks usually develop a stronger core-periphery structure (42), and many measures in this cluster could be related to this theme. Cluster 4 (“Popularity Norms Cluster”; *n* = 7) included features on student popularity based on student characteristics, e.g. socioeconomic background and grades. Finally, we identified two clusters that encapsulated indicators of homophily. Cluster 5 (“Social Homophily Cluster”; *n* = 4) pertained to social homophily, which examines whether students form friendships on the basis of shared social backgrounds, such as similar parental education levels. In contrast, Cluster 6, labeled the “Achievement Homophily Cluster” (*n* = 11 variables) focused on achievement-related homophily, analyzing the extent to which students befriended peers with similar academic performance levels. Five variables could not be clearly assigned to a cluster on an empirical basis, and multiplex networks were not included because they could not be assigned to a single network. It should be noted that our cluster labels provide only a rough characterization of the majority of, but not all, features within each cluster. To illustrate the type of network structures described by each cluster, Fig. 2 shows exemplary networks that differ in an example feature from each cluster.

### Control variables

To estimate the unique variance explained by the network features, we included a comprehensive list of control variables. These variables were sourced from both administrative information and parent questionnaires. From administrative records, we included classroom size, school track, gender ratio, and number of students with special educational needs. From the parent questionnaires, we incorporated several socioeconomic and demographic factors. These included the highest family socioeconomic index of occupational status (43), the highest family Erikson-Goldthorpe-Portocarero (EGP) class (44) and their variation, the average parental education level (according to the International Standard Classification of Education [ISCED] (45)), and its variation within the class. Furthermore, we accounted for the proportion of students with a first language other than German, the number of first languages spoken, the number of ethnic groups (identified via students’ own and their parents’ country of birth) in the classroom, and ethnic heterogeneity and language heterogeneity using Simpson's *D* (46). Finally, we included the classroom participation rate in the social network questionnaire as a control variable.

### Analyses

#### Elastic net regressions

To analyze the relationships between social network features and the correlates at the classroom level, we used elastic net regression analysis for predictive modeling (47). Model training and hyperparameter tuning were conducted in R using the caret package (48). The data were split into training (80%, *n* = 1,143) and testing (20%, *n* = 290) data at random. By performing out-of-sample validation of our results on the hold-out testing data, we effectively controlled for overfitting. Details on the model fitting process can be found in Supplement D.

#### Regression model specification

We finally fit (i) a model with only the 15 control variables described above (“Control variables only model”), (ii) a model including the control variables, friendship density, and centralization as “gold-standard” indicators (“Gold-standard model”), (iii) a model including the control variables and all network features (“All network features model”), (iv) a model including the control variables excluding school type and all network features (“Track excluded model,” reported in Table E1 in Supplement E), and (v) a model similar to (iii), additionally including all interactions between network features and school track (using a dummy for the highest track and a dummy for the lowest track, “Interactions model,” reported in Table E2 in Supplement E) because school tracks in Germany are often deemed profoundly different developmental contexts.

#### Model evaluation

In a first step, we evaluated the predictive power of the regression models by out-of-sample validation within the 20% hold-out data, thus safeguarding against type I errors and overfitting. Specifically, we compared the prediction root mean square error (RMSE) and proportion of variance explained (prediction *R*^2^) by the model that included only the control variables with the model that included the social network features. We applied this step for both the models that included school track and the model that excluded school track from the list of control variables (see Table E1 in Supplement E), as the allocation of students to school tracks was based on student achievement, and thus, this variable is causally determined by achievement to some extent. For comparison, we also present the baseline RMSE obtained by predicting the hold-out data using the mean of the training data (“Null model”), providing a benchmark for the predictive power of the control variables relative to this baseline. We determined the significance of the reduction in RMSE and the improvement in *R*^2^ by using a bootstrapping procedure with 1,000 iterations.

For outcomes for which adding the network features significantly improved the prediction, we proceeded to the second step of exploring the significance of the sets of features. Features were clustered in two different ways. First, five sets of indicators were built, each containing all the information from one network (friendship, break time, help, or rejection) or multiplex indicators (feature sets 1–5). Second, features within each feature set were clustered using k-medoids clustering (for details, see Supplement C).

To investigate the importance of features, we used SHapley Additive exPlanations (SHAP) values (49, 50), which specify the marginal average contribution of each variable for an outcome. We used a coalition size of three times the number of network features in the corresponding elastic net model to estimate the SHAP values. We summed up the absolute SHAP values across each set of features to determine the combined importance of these feature sets instead of interpreting individual features’ importance, which is not meaningful in elastic net models with many interrelated features (18). However, we also plotted the SHAP values for each feature for each classroom to see the direction of the effect of each predictor (see Supplement F for results).

#### Missing value treatment

**Figure 3:**
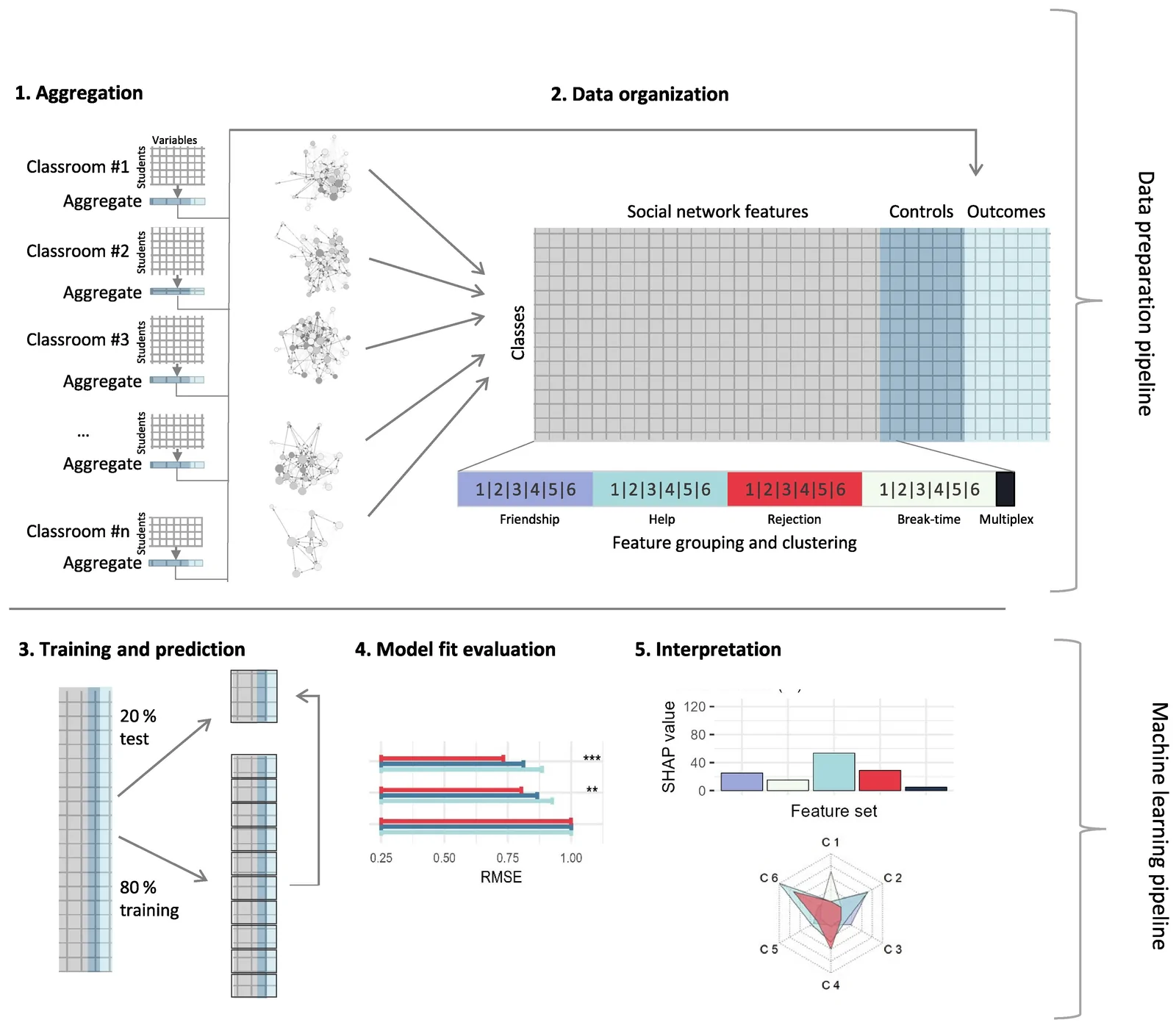
Data preparation and data analysis pipeline. (1) Aggregation of individual student data at the classroom level and computation of social network features. (2) Grouping of social network features by (i) network type (friendship, help, rejection, break time, and multiplex indicators) and (ii) empirical clusters (1–6). (3) Estimation of elastic net regression analyses for each of 33 outcomes using 20% hold-out data for validation of the final estimated model and 10-fold cross-validation for parameter tuning. (4) Determination of prediction accuracy (RMSE) and explanatory power (*R*^2^) of regression models. Selection of outcomes for further exploration of results. (5) SHAP-importance of social network feature groups and clusters.

We only used classrooms that had a minimum participation rate in the network questionnaire of 70%. After excluding the classrooms that did not meet this criterion, 1.0% of individual students had missing data in the network questionnaire. To deal with these missing values, we followed the reconstruction approach (51): Missing nominations of students were supplemented with their reciprocal value, i.e. if the other person nominated a student who had missing values for the network questionnaires, then the missing tie was supplemented with 1 (nomination). If a student was not nominated by another student, then the missing value was supplemented with 0 (no nomination). If both ties were missing, a value was imputed by drawing from a binomial distribution using the observed edge density of the current network as the probability parameter. Missing values in all other variables (outcome variables) were partly due to the planned missingness design of the study and could be considered to be missing completely at random (between 50 and 67% in achievement scores and 3% of academic climate variables). They resulted to some extent from students either not returning informed consent sheets or not being part of the sampling plan for the main study (17.5%). Individual item nonresponse varied for the variables but amounted to 1–4%. All missing values were handled with Bayesian stochastic regression imputation based on chained equations (52). For the imputation model, we chose the same set of features in our background model as in the original report of the Trends in Student Achievement Study (28) and added variables we used in our study that were not yet included. We ensured that the independent and dependent variables did not overlap in the predictor matrix so that the relationships between the dependent and independent variables (i.e. the control variables) in our study would not be inflated by the algorithm. We imputed the individual-level data of the training set first and then used the final imputation model to impute the values in the hold-out dataset, thereby maintaining full independence for model evaluation. The hold-out data comprised a random sample of 20% of the classrooms. Thus, the training set included 1,143 classrooms, and the hold-out data comprised 290 classrooms. Table A1 in Supplement A includes percentages of individual-level missing values for all variables in the study. The final datasets were computed by aggregating the scores on the classroom level across the imputed dataset. Figure 3 presents the schematic data preparation and analysis pipeline.

## AI disclosure statement

To support the preparation of this article, ChatGPT—5.5 (OpenAI, Inc.) was used for optimizing the wording and sentence structure of parts of the manuscript. In addition, ChatGPT was used for debugging of software code. All AI-generated suggestions were reviewed and edited by the authors prior to inclusion in the text. The authors take full responsibility for the content of this publication.

## Results

### Explanatory power of network characteristics

To evaluate the predictive models, we examined the reduction in RMSE and the increase in the explained variance by applying the estimated models to predict the dependent variables in a 20% hold-out dataset. In all the predictive modeling, we focused on the contributions of network indices after accounting for the control variables. The entire set of 314 network indicators was able to reduce the RMSE of the prediction by up to 0.11 standard deviations and explained up to 18% of the variance in the outcomes in the test data beyond the control variables in elastic net regressions (Table E1 in Supplement E). For each outcome variable considered, Fig. 1 shows the RMSE for three models: the “Null model” (i.e. using the mean of the training data for all classrooms in the test dataset), the “Control variables only model” (model 1), and the “All network features model” (model 2). Furthermore, the figure shows the outcomes for which the improvement in the RMSE and the explained variance (*R*^2^) were significant.

The results indicate that social network characteristics play a unique role in student functioning in addition to control variables in some domains, but not in others. Specifically, for students’ socio-emotional well-being (subjective social integration and well-being in school) and academic achievement (in mathematics, physics, and chemistry), network features significantly improved the prediction (RMSE and *R*^2^) beyond the control variables. However, for motivation, academic climate, teacher attunement, and instruction, we could not find improvement above the included control variables (see Fig. 1). Thus, we focused our subsequent analyses on the four significant outcome variables. See Supplement E for the full set of outcomes.

Unlike the current study, previous research has mostly used only a limited set of theoretically derived social network features to examine their relationships with outcome variables. In particular, studies often used density as a measure of the overall connectedness of the network and centralization as a measure of hierarchy (2, 15, 33–35, 53). To examine whether our predictive modeling approach was able to leverage information that could not be captured by these “gold-standard” network indices, we conducted an additional analysis with the limited set of commonly used network indicators (model 3, “Gold-standard analyses”). Specifically, we ran the same predictive models using friendship network density and centralization as the sole network feature predictors.

The “gold-standard” indicators significantly improved prediction over the control variables for subjective social integration and well-being in school (see Table 1 “Gold-standard analyses”). However, for four out of five focal outcome variables, adding the other network indices significantly improved the prediction, suggesting that the gold-standard network indices are not always sufficient for a full understanding of the relationships between social network structure and classroom outcomes (see the “Incremental predictive value” column in Table 1).

**Table 1:** Predictive power of network features beyond the control variables and the gold standard.

Outcome variable	Gold-standard analyses^a^		All network features^b^		Incremental predictive value^c^	
	ΔRMSE^d^	Δ*R*^2d^	ΔRMSE^d^	Δ*R*^2d^	ΔRMSE	Δ*R*^2^
Emotion						
Social integration (M)	0.055**	9.2%**	0.107***	17.6%***	0.052*	8.4%*
Social well-being (M)	0.028*	6.2%*	0.062**	11.0%**	0.028	4.9%
Academic achievement						
Chemistry (M)	<0.000	0.0%	0.018*	1.8%*	0.018*	1.8%*
Mathematics (M)	<0.000	0.0%	0.020**	2.0%*	0.020**	2.0%**
Physics (M)	0.001	0.0%	0.022*	2.0%*	0.021*	2.0%*

^a^Gold-standard analyses include control variables and two network features: friendship density and friendship centralization.

^b^All network features include control variables and all network features.

^c^The incremental predictive value column compares the model with all network features with the model with gold-standard features only.

^d^Gold-standard analyses and all network features were compared with a model with control variables only (not included in the table). Results were based on 290 classrooms in the hold-out data. Significance testing was performed using 1,000 bootstrap iterations. The reported significance levels indicate whether the bootstrapped 95, 99, or 99.9% CIs excluded zero. All tests were two-sided.

^*^ *P* < 0.05.

^**^ *P* < 0.01.

^***^ *P* < 0.001.

### Exploration of the relevance of different network features

While this first set of analyses was concerned with the extent to which the full set of network features could predict classroom outcomes, we pursued additional, more in-depth analyses to gain insight into which network features are especially predictive of social integration and social well-being as well as achievement in physics, chemistry, and mathematics. One challenge is that these network features are sometimes highly correlated with each other, and it is often difficult to pin down the contributions of individual network features (54). In fact, some network features embody similar information by definition (e.g. number of isolates and inclusiveness, see Supplement B), and it is not appropriate to simply evaluate and interpret the importance of individual network features when there are such redundancies.

To overcome this challenge, we evaluated the importance of groups of network indices that embody similar information, rather than individual predictors. We first examined the relative importance of four different types of social networks and multiplex features. Then, in a second analysis, we grouped the network features in a bottom-up, data-driven fashion. To investigate the importance of these groups of features, we aggregated SHAP values by summing them across feature groups (50). These group SHAP values capture the overall importance of feature clusters but not the direction of their effects. Therefore, while individual feature effects should not be overinterpreted in isolation, we use the individual SHAP distributions in Supplement F (Figs. F1–F4) to illustrate how the identified feature clusters relate to the regression outcomes.

#### Types of social networks

**Figure 4:**
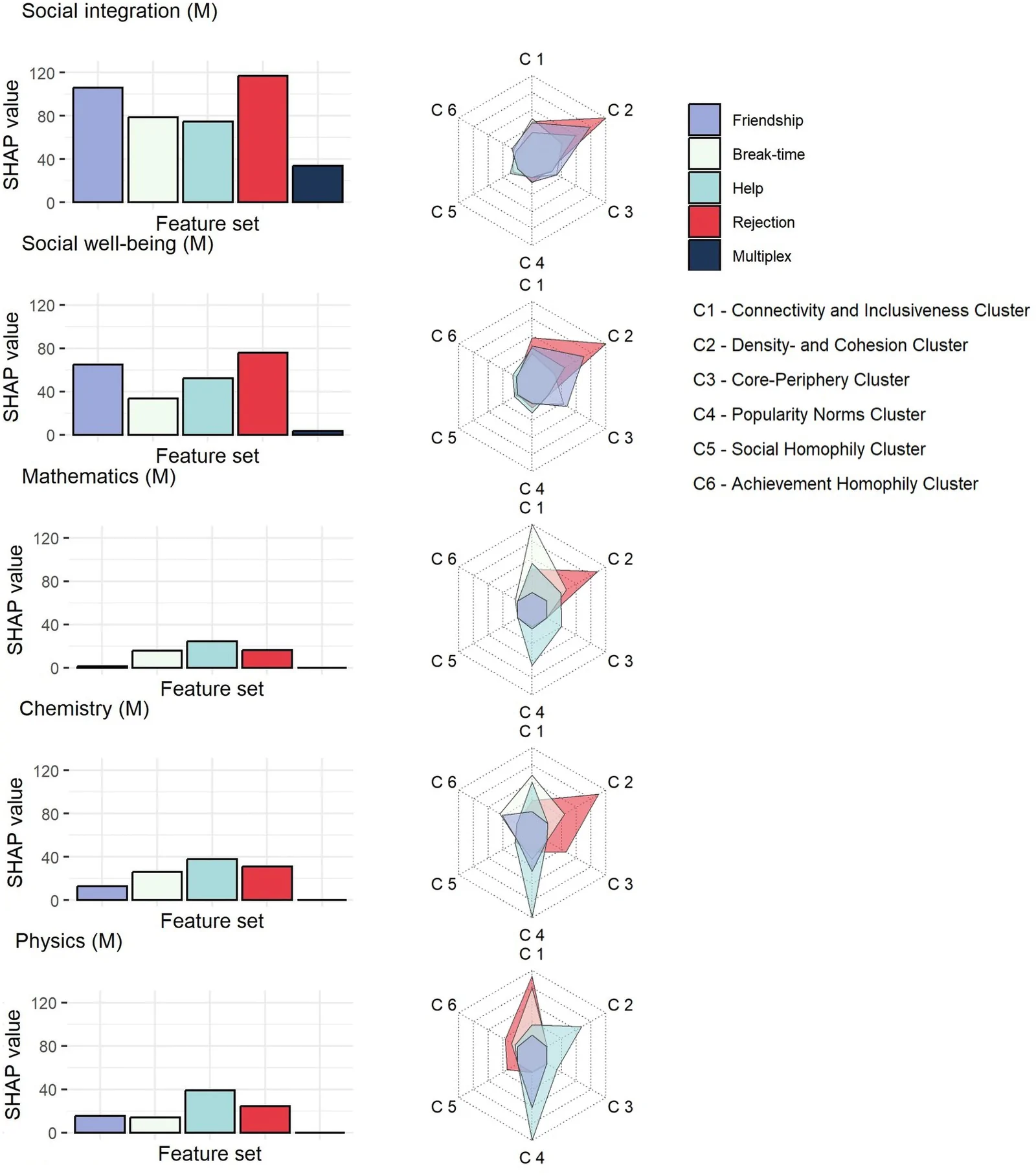
Aggregated feature importance by predictor set. Each row corresponds to one outcome. The total absolute SHAP value importance for all features within each of the networks (friendship, break time, help, rejection, and multiplex) is displayed on the left side. The SHAP value importance for the empirical clusters (C1–C6) within each of the four networks is depicted on the right side. Each network represents one classroom from the original data set.

Our data contain features calculated for each of four different social networks—friendship, break time, asking for help, and rejection as a deskmate—and features that are computed by combining these different aspects, called multiplex. To disentangle the importance of each of these network types, we decomposed the results of each prediction model into five importance scores. For each outcome, Fig. 4 (left) shows the absolute SHAP values summed up for the different social networks.

For competence in mathematics, chemistry, and physics, network indices computed from the help network (i.e. which classmates students turned to for help) were consistently very important for the outcomes. Similarly, network features capturing rejection as a deskmate and who spent time together during breaks were also important for all three outcomes, most pronounced for Chemistry. Friendship only exhibited some importance in chemistry and physics, but not in mathematics. By contrast, multiplex indicators did not contribute to predicting the results overall.

Different types of networks played more significant roles in socio-emotional outcomes in education (subjective social integration and socio-emotional well-being in school) in comparison with academic achievement. Specifically, friendship and rejection networks were the strongest contributors, followed by the help network and the break-time network. Finally, the multiplex network also played a role, in contrast to the achievement outcomes.

#### Clusters of social network features

We also pursued a data-driven approach to group the network features, which resulted in six mathematical feature clusters. Figure 4 (right) shows the absolute SHAP values for the different social network types as well as the values broken up by feature clusters. For the academic achievement outcomes, there were consistent effects pointing to the elevated importance of the Popularity Norms Cluster in the help network. In mathematics, the popularity of mathematics high achievers was uniquely associated with higher performance, while in physics and chemistry, the popularity of high achievers in chemistry emerged as an additional important feature (see Fig. F3 in Supplement F). Also, the break time and help networks’ Connectivity and Inclusion Cluster proved to be important in all three subjects. In particular, higher out-degree inclusiveness (few students that nominate no peers) and a low disparity in nomination behavior were associated with better academic outcomes. For both mathematics and chemistry, the rejection Density and Cohesion Cluster were similarly important, in contrast to physics, where only the Connectivity and Inclusion Cluster of this network was predictive.

With the cluster-specific analyses for the socio-emotional outcomes, we found similar results for social integration and social well-being. Specifically, the features signifying the Density and Cohesion Cluster consistently had the highest aggregated importance in the friendship and rejection networks. Inspection of individual SHAP values (Fig. F4) showed that the existence of multiple smaller, highly dense friendship cliques was associated with more positive socio-emotional outcomes. In the rejection network, a low proportion of empty triads and a higher prevalence of unstable triadic configurations were associated with a poorer socio-emotional climate. These triads were characterized by inconsistent patterns, in which mutually rejecting dyads would show alignment in rejecting a third person (which goes against the idea that “the enemy of my enemy is my friend”). Only social well-being was related to the Core-Periphery Cluster.

## Discussion

There has been a vast amount of literature on predicting academic outcomes from individual social experiences and relationships, such as being popular, accepted by others, and having friends; giving and receiving social support; or being rejected or excluded by peers (6, 55–59). However, we aimed to determine whether it was possible to predict variance in academic outcomes by using “solely macro-level” social network information (i.e. classroom peer ecology information) in classrooms. To do so, we examined the power of more than 300 social network features to predict a broad set of 33 classroom characteristics. Leveraging machine-learning methods and the largest German social network dataset of 1,433 secondary school classes, we showed that social network features can indeed significantly improve prediction over a host of control variables for a selective set of school outcomes, namely, social well-being in school, subjective social integration, and academic achievement in mathematics and chemistry. However, for academic climate, motivation and teacher-related outcomes, network indices did not significantly improve prediction. While subjective social integration and sociometric measures are expected to be related to some extent, they represent conceptually and empirically distinct constructs (58, 60, 61). At the individual level, associations between students’ subjective perceptions of social integration and their peer nominations were at most medium-sized in the current study (see Table A5 in Supplement A). Moreover, the predictors examined in the present analysis capture emergent structural characteristics at the classroom level. As such, they reflect global properties of classroom network structure rather than simple aggregations of individual ratings.

The additional variance explained by the network features amounted to up to 18% for socio-emotional outcomes and up to 2% for achievement outcomes after we controlled for a large number of background variables. According to benchmarks, these values can be interpreted as medium to large for socio-emotional outcomes and small to medium for achievement outcomes (62). The improvement beyond the control variables is noteworthy, especially because achievement in Germany has been found to be determined to a large extent by family background, school track, and prior achievement. For example, Lavelle-Hill et al. (18) showed that 75% of the variance in grade 9 achievement was explained only by school track and prior performance. The prediction improved by only a 2% increase in explained variance when they added grades, family background information, cognitive tests, and self-reports on motivation, emotion, and instruction. Thus, our findings indicate that classroom social structure is an additional independent factor in a successful academic career.

Our results also indicate that the explanatory power of the peer ecology depends on the type of network (e.g. friendship and asking for help). Indeed, our findings are consistent with the expectations a person might hold: Whereas the network of helping relationships between students was most strongly related to academic outcomes, socio-emotional outcomes were best predicted by friendship and rejection. In contrast, the friendship network did not play a major role in explaining academic outcomes. We are aware of only a little research that has directly compared the importance of different network types for academic or psychosocial outcomes. However, these results are consistent with existing theories. For example, attachment theory (63) indicates that experiences of friendship and rejection represent students’ psychosocial adaptation (64), supporting our findings that socio-emotional well-being and subjective integration are predicted by friendship and rejection networks. In social constructivist theories (3, 11), on the other hand, cognitive development is related to diverse cognitive-instrumental peer relationships, reinforcing our results on the heightened significance of helping relationships for academic achievement. At the same time, we find no evidence that multiplex network structure is associated with academic achievement outcomes. From this perspective, it appears less important whether cognitively instrumental ties are, for example, embedded within friendship relations than whether such help-seeking relations are present at all. Accordingly, the degree to which different types of peer relations co-occur (i.e. overlap or correlation between friendship and help networks) does not appear to be systematically related to achievement differences between classes. Instead, achievement is more closely linked to the presence of functionally relevant relations, such as help-seeking ties. One implication from these findings is that researchers should pay more attention to the type of social network, considering the educational outcomes they are interested in, as empirical results could be substantially different depending on the type of network researchers’ use.

One interesting observation from our study is that popularity norm-related features were central to academic achievement, that is, the classrooms in which students with better grades were asked for help more often (high correlation between student achievement and in-degree centrality in a classroom) showed better achievement levels overall. This finding lines up with theoretical work linking norm salience, i.e. the degree to which sociometric choices are related to certain behaviors, with the prevalence of this behavior in a group (person-group similarity model, e.g. Verschueren et al. (65); social context model (14)). These theories propose that a behavior that is common in a context is also valued positively, and students who do not conform to this behavior face rejection. For example, in schools in the highest track, students typically show high achievement, and high achievers are also more popular than in lower track schools (66).

With more than 300 network metrics, we identified six correlation-based clusters. The cluster structure was relatively consistent across different types of networks, indicating that these clusters represent robust groups of network metrics. These clusters represented important social network characteristics identified by the previous social network literature. For example, there is a body of research on the relationship between network structure and team performance in organization science (67). In these studies, there has been a focus on “efficient” networks that are well-connected and facilitate the spread of information across actors (68). However, prior empirical research has often excluded disconnected networks, and thus, the network features that we found to be important (e.g. isolation, inclusiveness, and components) were not represented in this research. Instead, network metrics used in prior studies have relied largely on indicators related to those in our “Density and Cohesion Cluster.” Our analyses showed that these are distinct themes and that organizational research could profit from including features from the “Connectivity and Inclusiveness Cluster.” Another cluster that lined up with earlier research was our “Core-Periphery Cluster.” The core-periphery structure of networks is characterized by a dense core with less well-connected nodes in the periphery. This type of network has been found in molecular and cellular networks, ecological, social, and economic networks (41). Yet, classroom social networks have not yet been described in terms of these distinct clusters. Future research should examine the replicability of the cluster structure that we identified in the current work and should test whether the clusters represent important and generalizable properties of classroom social networks in schools. In confirming the relevance of specific network clusters for specific outcomes, research will be able to identify promising targets of network-informed interventions (69).

While our work demonstrated the value of the peer ecology for understanding factors related to academic outcomes in school, the cross-sectional nature of the study does not permit causal inferences about the relationship between network structures and educational outcomes. In fact, the relationship we identified in the current study is likely to reflect co-evolutionary, bidirectional processes. For example, individual students with their specific characteristics interact with each other, and these individual interactions can constitute relationships that give rise to social network structures (70, 71). These structures can then in turn become factors in shaping the way students interact (70, 72). Given the study's primary focus on structure rather than processes, we leave it to future research to elucidate the mechanisms through which these macro-level structures emerge from individual-level interactions and through which pathways they influence individual student outcomes (73). These mechanistic and developmental perspectives pave the way for targeted, network-informed interventions that enhance both educational outcomes and students’ social lives in schools.

Overall, our analyses highlighted the role of the classroom peer ecology to predict learning and well-being in school: Our findings indicate that network features are effective tools for capturing essential aspects of social functioning that go beyond the traditional background variables that are often included as a measure of social mixing. At the same time, the social network features did not improve prediction of other aspects of the educational experience of students, such as the instructional methods used or motivational aspects. It remains to be seen whether longitudinal and experimental designs will corroborate these findings. Future research should explore the generalizability of our findings by including student samples from different countries and educational stages. While the importance of human relationships is ubiquitous, we expect that individualist and collectivist cultures will differ in how children interact, which network structures they form, and how these social structures are related to student outcomes (74). Just as students are embedded in classrooms, schools and classrooms are also embedded in their broader cultural and economic contexts. Examining the classroom peer ecology within this larger structure can provide a more comprehensive understanding of how to support the long-term development of schoolchildren and foster greater social cohesion.

## Supplementary Material

pgag291_Supplementary_Data

## Data Availability

The data we used in this study are available from the Research Data Centre at the Institute for Educational Quality Improvement at http://doi.org/10.5159/IQB_BT_2018_v1. The code to reproduce the analyses in this paper is freely available at https://osf.io/8qhuw/?view_only=a33c7db6e6c24fff868e6990d3d6da7e.

## Notes

a In the analyses reported in the original manuscript, classrooms were included if at least 25% of students provided network data. Following a reviewer’s suggestion, we adopted a more conservative inclusion criterion requiring data from at least 70% of students, ensuring that network measures were based on sufficiently complete classroom networks. Although this reduced the number of classrooms available for analysis by 470, thereby reducing statistical power, it also reduced the risk of bias arising from the tie imputation procedure, which may become less reliable at higher levels of missingness. Results obtained using the original 25% threshold are reported in Supplement G and were largely similar to those presented here.

## Preprints

The preprint is deposited at https://osf.io/preprints/psyarxiv/krcdm_v1.
