# Supplementary material for: The global structure of classroom social networks predicts academic and psychosocial adjustment
Source: PNAS Nexus. 2026 Aug 26;5(9):pgag291. doi: 10.1093/pnasnexus/pgag291 (PMC13591403; doi:10.1093/pnasnexus/pgag291)
Supplement: Supplementary file 1 [file pgag291_supplementary_data.zip › PNASNEXUS-PNASNEXUS-2025-01871RR-s01.pdf]

SUPPLEMENTS

Supplement A (Descriptive Information) 2
Supplement B (Network features list) 14
Supplement C (Clustering procedure) 15
Supplement D (Estimation procedure of the elastic net regression models) 16
Supplement E (Explanatory power for all outcomes) 18
Supplement F (Individual Feature Importance) 26
Supplement G (Robustness) 32

Supplement A (Descriptive Information)

Table A1. Sample descriptives per academic track

Variable	Highest track	Intermediate tracks	Lowest track	Total	Missing
Nstudent	13830	20080	1128	35038	0.0%
Nclass	551	828	54	1433	0.0%
Gender (% girls)	52.1%	47.0%	39.9%	48.8%	1.0%
Age (years)	15.38 (0.51)	15.69 (0.69)	15.85 (0.75)	15.57 (0.65)	0.5%
Socio-economic status (ISEI)	61.2 (18.82)	46.59 (18.96)	41.01 (18.01)	52.45 (20.27)	15.1%
Native german (%)	88.4%	81.5%	67.8%	83.8%	3.9%
Competences					
Mathematics (M)	0.95 (1.06)	-0.48 (1.09)	-0.85 (1)	0.08 (1.29)	49.1%
Physics (M)	0.82 (1.22)	-0.44 (1.23)	-0.98 (1.21)	0.05 (1.38)	65.0%
Chemistry (M)	0.74 (1.13)	-0.45 (1.17)	-1.03 (1.05)	0.01 (1.3)	65.0%
Biology (M)	0.76 (1.17)	-0.45 (1.17)	-0.95 (1.1)	0.02 (1.31)	66.2%
Motivation					
Interest biology (M)	2.63 (0.76)	2.44 (0.77)	2.27 (0.79)	2.51 (0.77)	11.9%
Interest chemistry (M)	2.28 (0.83)	2.26 (0.81)	2.18 (0.83)	2.27 (0.82)	12.1%
Interest physics (M)	2.24 (0.83)	2.19 (0.8)	2.15 (0.84)	2.21 (0.81)	12.1%
Interest mathematics (M)	2.31 (0.79)	2.29 (0.78)	2.38 (0.85)	2.3 (0.79)	11.4%
Mathematics anxiety (M)	2.16 (0.8)	2.29 (0.81)	2.32 (0.83)	2.24 (0.81)	11.8%
Emotion					
Social integration (M)	3.31 (0.46)	3.19 (0.53)	3.17 (0.56)	3.23 (0.51)	13.0%
Social well-being (M)	3.22 (0.48)	3.12 (0.54)	3.1 (0.58)	3.16 (0.52)	13.6%
Academic climate					
Discipline mathematics (M)	2.21 (0.8)	2.35 (0.85)	2.36 (0.82)	2.29 (0.83)	13.7%
High standards (M)	2.38 (0.56)	2.18 (0.56)	2.06 (0.58)	2.26 (0.57)	13.5%
Boredom mathematics (M)	2.12 (0.76)	2.18 (0.8)	2.18 (0.85)	2.15 (0.78)	15.6%
Gender stereotypes students	3.17 (0.88)	3.16 (0.97)	3.34 (1.04)	3.17 (0.94)	14.8%
Social networks					
Friendship in-degrees	10.19 (4.04)	10.15 (4.22)	9.13 (3.64)	10.13 (4.14)	1.0%
Friendship out-degrees	10.19 (5.49)	10.15 (5.8)	9.13 (5.27)	10.13 (5.66)	1.0%
Contact in-degrees	5.49 (2.83)	4.74 (2.7)	4.23 (2.39)	5.02 (2.77)	1.0%
Contact out-degrees	5.49 (3.62)	4.74 (3.56)	4.23 (3.3)	5.02 (3.6)	1.0%
Help in-degrees	5.04 (3.07)	4.44 (2.79)	3.72 (2.35)	4.65 (2.91)	1.0%
Help out-degrees	5.04 (4.44)	4.44 (4.28)	3.72 (3.61)	4.65 (4.34)	1.0%
Rejection in-degrees	4.34 (3.26)	5.59 (3.47)	4.97 (2.85)	5.07 (3.43)	1.0%
Rejection out-degrees	4.34 (4.41)	5.59 (5.17)	4.97 (4.69)	5.07 (4.9)	1.0%

Note. The table includes data from our analytic sample. Missing values in competence scores were planned due to the booklet design and can be considered largely missing completely at random (MCAR). Similarly, 2.8% of the missing values in the climate variables were due to the booklet design. 17.5% of the students did not provide informed consent to participate in the voluntary questionnaire and were therefore missing the complete questionnaire data. Other missing values were due to individual student non-response.

Table A2. Classroom-level sample descriptives

Variable	Highest track	Intermediate track	Lowest track	Total
Nclass	551	828	54	1433
Class size	25.10 (3.81)	24.25 (4.24)	20.89 (4.23)	24.45 (4.16)
Competences				
Mathematics (SD)	0.93 (0.16)	0.92 (0.17)	0.89 (0.15)	0.93 (0.17)
Physics (SD)	1.01 (0.19)	1.02 (0.19)	0.98 (0.19)	1.01 (0.19)
Chemistry (SD)	1 (0.19)	1.04 (0.18)	0.97 (0.21)	1.02 (0.19)
Biology (SD)	1.07 (0.19)	1.11 (0.19)	1.10 (0.19)	1.09 (0.19)
Mathematics (M)	0.92 (0.49)	-0.52 (0.58)	-0.90 (0.56)	0.02 (0.90)
Physics (M)	0.74 (0.5)	-0.51 (0.59)	-0.97 (0.57)	-0.05 (0.84)
Chemistry (M)	0.7 (0.49)	-0.52 (0.56)	-1.03 (0.5)	-0.07 (0.81)
Biology (M)	0.78 (0.52)	-0.48 (0.58)	-0.97 (0.59)	-0.02 (0.85)
Academic climate				
Discipline Mathematics (M)	2.2 (0.54)	2.36 (0.5)	2.38 (0.38)	2.30 (0.52)
High standards (M)	2.39 (0.23)	2.17 (0.22)	2.06 (0.21)	2.25 (0.25)
Boredom Mathematics (M)	2.12 (0.26)	2.19 (0.27)	2.19 (0.26)	2.16 (0.27)
Gender stereotypes students	3.17 (0.43)	3.16 (0.41)	3.33 (0.41)	3.17 (0.42)
Emotion				
Social Integration (M)	3.31 (0.14)	3.18 (0.18)	3.17 (0.15)	3.23 (0.17)
Social Well-being (M)	3.21 (0.15)	3.12 (0.18)	3.10 (0.14)	3.15 (0.18)
Teacher Stress (M)	2.49 (0.3)	2.52 (0.33)	2.41 (0.37)	2.50 (0.32)
Teacher attitudes				
Gender stereotypes teacher	3.08 (0.27)	3.1 (0.29)	3.33 (0.39)	3.10 (0.29)
Importance fostering social competences	3.24 (0.38)	3.3 (0.37)	3.46 (0.45)	3.28 (0.38)
Fostering joy of learning and social integration	3.08 (0.4)	3.17 (0.42)	3.22 (0.50)	3.14 (0.42)

Table A2 (continued)

Variable	Highest track	Intermediate track	Lowest track	Total
Instruction				
Small group instruction	2.86 (0.47)	2.79 (0.52)	2.96 (0.61)	2.82 (0.51)
Individual study	3.22 (0.45)	3.26 (0.45)	3.49 (0.45)	3.25 (0.46)
Whole classroom instruction	3.55 (0.35)	3.45 (0.41)	3.62 (0.53)	3.49 (0.40)
Interdisciplinary learning	2.26 (0.52)	2.37 (0.53)	2.35 (0.7)	2.33 (0.54)
Free study time	1.65 (0.44)	1.89 (0.56)	2.13 (0.67)	1.8 (0.54)
Peer tutoring	1.75 (0.49)	1.83 (0.58)	2.08 (0.77)	1.81 (0.56)
Weekly plan	1.26 (0.36)	1.55 (0.58)	1.88 (0.9)	1.45 (0.54)
Project-based learning	1.74 (0.4)	1.82 (0.42)	1.83 (0.61)	1.79 (0.42)
Heterogenous grouping	2.80 (0.58)	2.85 (0.55)	3.03 (0.52)	2.84 (0.57)
Homogenous grouping	2.40 (0.54)	2.50 (0.48)	2.51 (0.62)	2.46 (0.51)
Student motivation				
Interest Biology (M)	2.63 (0.25)	2.43 (0.27)	2.28 (0.27)	2.50 (0.29)
Interest Chemistry (M)	2.28 (0.29)	2.25 (0.28)	2.19 (0.23)	2.26 (0.28)
Interest Physics (M)	2.24 (0.29)	2.19 (0.28)	2.16 (0.26)	2.21 (0.28)
Interest Mathematics (M)	2.30 (0.23)	2.28 (0.22)	2.39 (0.24)	2.29 (0.23)
Mathematics Anxiety (M)	2.17 (0.22)	2.30 (0.23)	2.33 (0.2)	2.25 (0.24)
Social networks				
Friendship average degree	20.10 (4.57)	19.82 (5.32)	17.77 (4.65)	19.85 (5.04)
Friendship density	0.43 (0.1)	0.44 (0.11)	0.46 (0.12)	0.43 (0.1)
Contact average degree	10.86 (3.01)	9.31 (2.91)	8.23 (2.53)	9.87 (3.04)
Contact density	0.23 (0.07)	0.21 (0.07)	0.21 (0.06)	0.22 (0.07)
Help average degree	9.95 (2.59)	8.67 (2.78)	7.23 (2.26)	9.10 (2.78)
Help density	0.21 (0.05)	0.19 (0.06)	0.19 (0.06)	0.20 (0.06)
Rejection average degree	8.53 (2.99)	10.86 (4.19)	9.57 (3.5)	9.92 (3.91)
Rejection density	0.18 (0.06)	0.24 (0.08)	0.24 (0.07)	0.22 (0.08)

Table A3. Description of network features used in the analysis and their means and standard deviations

Cluster	varname	Feature name	M friend (SD)	M help (SD)	M contact (SD)	M rejection (SD)	M multi (SD)
1	cohesion_directed	cohesion (minimum number of students to remove to split the graph into disconnected subgraphs)	1.45 (1.39)	0.07 (0.27)	0.18 (0.48)	0.04 (0.24)	
1	component_ratio_s	component ratio (strongly connected subgraphs by classroom size)	0.02 (0.04)	0.17 (0.13)	0.11 (0.10)	0.24 (0.16)	
1	component_ratio_w	component ratio (weakly connected subgraphs by classroom size)	0.00 (0.01)	0.02 (0.04)	0.02 (0.04)	0.02 (0.05)	
1	components_strong	components (number of strongly unconnected subgraphs)	1.42 (0.86)	4.95 (2.84)	3.57 (2.25)	6.51 (3.83)	
1	components_weak	components (number of weakly unconnected subgraphs)	NA	1.42 (0.84)	1.50 (0.87)	1.45 (1.07)	
1	connectedness	connectedness (average component size by classroom size)	0.82 (0.25)	0.29 (0.22)	0.41 (0.27)	0.23 (0.18)	
1	CV_in	coefficient of variation for out-degree	0.34 (0.10)	0.57 (0.15)	0.47 (0.13)	0.60 (0.21)	
1	CV_out	coefficient of variation for in-degree	0.51 (0.10)	0.86 (0.19)	0.64 (0.15)	0.92 (0.24)	
1	equity_in	network equity (coefficient of variation of students' degree centrality)	-0.34 (0.10)	-0.57 (0.15)	-0.47 (0.13)	-0.60 (0.21)	
1	in.isol	number of isolated students	0.09 (0.49)	0.95 (1.34)	0.67 (1.07)	0.92 (1.68)	
1	in.isol_inclusiveness	inclusiveness (proportion of students included in a classroom)	1.00 (0.02)	0.96 (0.06)	0.97 (0.05)	0.96 (0.07)	
1	out.isol	Number of students who did not nominate any classmate	0.31 (0.57)	2.71 (1.92)	1.45 (1.37)	4.43 (2.89)	
1	out.isol_inclusiveness	Proportion of students who nominated at least one classmate	0.99 (0.02)	0.89 (0.08)	0.94 (0.06)	0.82 (0.11)	

Cluster	varname	Feature name	M friend (SD)	M help (SD)	M contact (SD)	M rejection (SD)	M multi (SD)
1	triad_cen_3	proportion of specific triads	0.23 (0.07)	0.13 (0.04)	0.25 (0.07)	0.09 (0.04)	
2	avg_distance	average distance between any two students in the classroom	1.66 (0.19)	2.27 (0.33)	2.35 (0.42)	2.04 (0.29)	
2	centralization_fm	Freeman centralization	0.27 (0.07)	0.26 (0.09)	0.20 (0.07)	0.31 (0.12)	
2	clust_coeff	clustering coefficient	0.72 (0.08)	0.56 (0.12)	0.63 (0.12)	0.48 (0.13)	
2	cohesion_undirected	cohesion (considering nominations as undirected)	4.16 (2.48)	1.29 (1.18)	1.20 (1.22)	1.73 (1.57)	
2	compactness	average reciprocal of geodesic distances between each pair of students	0.76 (0.07)	0.60 (0.09)	0.56 (0.10)	0.63 (0.10)	
2	coreness_all_min	minimal coreness of vertices in a class (for each student, max subgraph in which every vertex had as least k degrees)	6.06 (3.57)	1.59 (1.45)	1.97 (1.85)		
2	density	density of the network (proportion of existing ties)	0.43 (0.10)	0.20 (0.06)	0.22 (0.07)	0.22 (0.08)	
2	diameter	diameter (longest path between two students)	3.47 (0.75)	5.31 (1.27)	5.59 (1.45)	4.42 (1.03)	
2	max_1Clan	1Clan	0.40 (0.10)	0.24 (0.06)	0.27 (0.07)	0.22 (0.06)	
2	max_2Clan	2Clan	0.93 (0.10)	0.75 (0.18)	0.61 (0.18)	0.81 (0.16)	
2	Segmentation_3	Proportion of indirect ties that are more than 2 steps long from all indirect ties	0.06 (0.09)	0.25 (0.20)	0.44 (0.22)	0.17 (0.18)	
2	triad_cen_1	proportion of specific triads	0.13 (0.08)	0.39 (0.11)	0.38 (0.12)	0.37 (0.14)	
2	triad_cen_2		0.13 (0.05)	0.25 (0.04)	0.19 (0.05)	0.24 (0.05)	
2	triad_cen_4		0.03 (0.02)	0.04 (0.03)	0.02 (0.02)	0.05 (0.03)	
2	triad_cen_5		0.01 (0.01)	0.02 (0.01)	0.01 (0.01)	0.03 (0.02)	
2	triad_cen_6	proportion of specific triads	0.01 (0.01)	0.02 (0.01)	0.01 (0.01)	0.03 (0.01)	

Cluster	varname	Feature name	M friend (SD)	M help (SD)	M contact (SD)	M rejection (SD)	M multi (SD)
2	triad_cen_7		0.04 (0.02)	0.02 (0.01)	0.02 (0.01)	0.02 (0.01)	
2	triad_cen_8		0.10 (0.03)	0.05 (0.03)	0.04 (0.03)	0.06 (0.04)	
2	triad_cen_9		0.01 (0.02)	0.02 (0.01)	0.01 (0.01)	0.02 (0.02)	
2	triad_cen_10		0.00 (0.00)	0.00 (0.00)	0.00 (0.00)	0.00 (0.00)	
2	triad_cen_11		0.09 (0.03)	0.02 (0.01)	0.02 (0.02)	0.03 (0.03)	
2	triad_cen_12		0.02 (0.01)	0.01 (0.01)	0.01 (0.01)	0.00 (0.00)	
2	triad_cen_13		0.03 (0.02)	0.01 (0.01)	0.01 (0.01)	0.01 (0.01)	
2	triad_cen_14		0.01 (0.01)	0.00 (0.00)	0.00 (0.00)	0.01 (0.01)	
2	triad_cen_15		0.08 (0.04)	0.01 (0.01)	0.02 (0.02)	0.02 (0.02)	
2	triad_cen_16		0.08 (0.06)	0.01 (0.01)	0.02 (0.02)	0.01 (0.01)	
3	asym	number of asymmetrical dyads	57.95 (25.08)	56.35 (24.43)	37.85 (18.75)	64.35 (28.07)	
3	centralization_gr_all	Gest and Rodkin Centralization for total degree	56.06 (24.96)	28.65 (16.25)	23.70 (15.80)	35.65 (19.21)	
3	centralization_gr_in	Gest and Rodkin Centralization for in-degree	11.04 (5.11)	6.61 (3.52)	5.43 (3.27)	7.93 (4.79)	
3	centralization_gr_out	Gest and Rodkin Centralization for out-degree	25.83 (12.70)	16.75 (11.39)	10.67 (8.32)	20.07 (11.88)	
3	cliques	size of the largest clique	9.51 (2.21)	5.74 (1.31)	6.50 (1.61)	5.11 (1.25)	
3	closeness_m_ug	average harmonic centrality of students	17.57 (3.23)	13.97 (3.16)	12.91 (3.14)	14.60 (3.41)	
3	coreness_all_sd	standard deviation of coreness of vertices in a classroom (max subgraph in which every vertex has as least degree k)	2.66 (1.18)	1.75 (0.59)	2.18 (0.91)	1.83 (0.62)	
3	embeddedness	number of cohesive blocks embedded within each other	4.39 (1.40)	4.76 (1.24)	4.82 (1.35)	4.58 (1.25)	

Cluster	varname	Feature name	M friend (SD)	M help (SD)	M contact (SD)	M rejection (SD)	M multi (SD)
3	mut	Number of reciprocated ties	93.67 (35.30)	28.12 (13.12)	41.86 (18.05)	29.18 (18.99)	
4	TR_NOTE_BIO_r2_centrality	centrality of students with a high grade, i.e., correlation between grade and in-degree	-0.10 (0.24)	-0.33 (0.24)	-0.08 (0.24)	0.15 (0.24)	
4	TR_NOTE_CHE_r2_centrality		-0.09 (0.23)	-0.33 (0.24)	-0.09 (0.25)	0.14 (0.24)	
4	TR_NOTE_DEU_r_centrality		-0.13 (0.23)	-0.36 (0.23)	-0.08 (0.25)	0.18 (0.23)	
4	TR_NOTE_MAT_r_centrality		-0.08 (0.23)	-0.32 (0.24)	-0.08 (0.23)	0.13 (0.23)	
4	TR_NOTE_PHY_r2_centrality		-0.09 (0.24)	-0.31 (0.24)	-0.09 (0.24)	0.13 (0.24)	
4	HISCED_centrality	centrality of students' parents' education	0.02 (0.22)	0.05 (0.21)	0.03 (0.22)	-0.02 (0.22)	
4	HISEI_centrality	centrality of students' socio-economic background	0.02 (0.22)	0.05 (0.22)	0.03 (0.21)	-0.02 (0.22)	
5	HISCED_assortativity	assortativity of students' parents' education	-0.04 (0.06)	-0.05 (0.10)	-0.04 (0.11)	-0.07 (0.10)	
5	HISEI_assortativity	assortativity of students' socio-economic background	-0.03 (0.09)	-0.02 (0.13)	-0.02 (0.14)	-0.04 (0.12)	
5	HISCED_moranI	Moran's I index of students' parental education	-0.04 (0.06)	-0.04 (0.10)	-0.04 (0.12)	-0.07 (0.10)	
5	HISEI_moranI	Moran's I index of students' socio-economic background	-0.03 (0.09)	-0.02 (0.13)	-0.02 (0.14)	-0.04 (0.12)	
6	TR_NOTE_BIO_r2_assortativity	homophily between students in the same grades	0.00 (0.09)	0.02 (0.13)	0.04 (0.15)	-0.10 (0.11)	
6	TR_NOTE_CHE_r2_assortativity		0.00 (0.09)	0.01 (0.13)	0.04 (0.15)	-0.10 (0.11)	
6	TR_NOTE_DEU_r_assortativity		0.01 (0.09)	0.02 (0.13)	0.05 (0.15)	-0.10 (0.11)	
6	TR_NOTE_MAT_r_assortativity		-0.01 (0.08)	0.00 (0.12)	0.01 (0.14)	-0.09 (0.11)	
6	TR_NOTE_PHY_r2_assortativity		-0.00 (0.08)	0.01 (0.13)	0.03 (0.14)	-0.09 (0.11)	
6	TR_NOTE_BIO_r2_moranI	Moran's I index of spacial autocorrelation, i.e., the extent to which close students were similar in grades	0.03 (0.12)	0.07 (0.17)	0.07 (0.17)	-0.06 (0.13)	
6	TR_NOTE_CHE_r2_moranI		0.03 (0.11)	0.07 (0.16)	0.07 (0.17)	-0.06 (0.13)	
6	TR_NOTE_DEU_r_moranI		0.04 (0.12)	0.07 (0.16)	0.08 (0.17)	-0.06 (0.13)	

Cluster	varname	Feature name	M friend (SD)	M help (SD)	M contact (SD)	M rejection (SD)	M multi (SD)
6	TR_NOTE_MAT_r_moranI		0.01 (0.10)	0.05 (0.16)	0.04 (0.16)	-0.06 (0.13)	
6	TR_NOTE_PHY_r2_moranI		0.02 (0.11)	0.06 (0.16)	0.06 (0.16)	-0.06 (0.13)	
6	in_degrees_assortativity	assortativity coefficient (in-degree)	0.01 (0.13)	-0.04 (0.14)	0.15 (0.21)	-0.19 (0.13)	
multiplex	degree_overlap_close_neg	Overlap of ties between different networks					0.62 (0.09)
multiplex	degree_overlap_friend_close						0.74 (0.08)
multiplex	degree_overlap_friend_help						0.69 (0.08)
multiplex	degree_overlap_friend_neg						0.44 (0.10)
multiplex	degree_overlap_help_close						0.81 (0.05)
multiplex	degree_overlap_help_neg						0.63 (0.09)
multiplex	degree_correlation_close_neg	Correlation of out-degree between different networks					0.02 (0.24)
multiplex	degree_correlation_friend_close						0.40 (0.22)
multiplex	degree_correlation_friend_help						0.28 (0.23)
multiplex	degree_correlation_friend_neg						-0.07 (0.24)
multiplex	degree_correlation_help_close						0.33 (0.24)
multiplex	degree_correlation_help_neg						0.06 (0.24)
	avg_degree	average number of nominations per student	19.85 (5.04)	9.10 (2.78)	9.87 (3.04)	9.92 (3.91)	
	closeness_m	average closeness centrality of students	0.03 (0.01)	0.03 (0.02)	0.04 (0.04)	0.03 (0.02)	
	CV_all	coefficient of variation for the total number of degrees	0.38 (0.08)	0.58 (0.11)	0.48 (0.11)	0.62 (0.15)	
	in_degrees_moranI	Moran's I index of students' in-degree	0.08 (0.17)	0.09 (0.17)	0.26 (0.22)	-0.10 (0.20)	
	reciprocity	Proportion of reciprocated ties	0.62 (0.09)	0.34 (0.09)	0.53 (0.12)	0.30 (0.12)	

Table A4. Wordings of items used in this study

Variable	Wording
	Socio-emotional outcomes
Social integration in the classroom	
	Other students seem to like me.
	I have many friends in my class.
	I spend my breaks with other students.
	I am often teased by other students. (rec.)
	I like most students in my class.
	Others enjoy spending time with me.
	I feel lonely during breaks. (rec.)
Well-being at school	
	I often feel like an outsider at school. (rec.)
	In this school, it is easy to make friends.
	I feel like I belong at this school.
	I often feel uncomfortable and out of place at this school. (rec.)
	I am liked by other students in this school.
	I feel lonely at this school. (rec.)
	I feel happy at this school.
	Things are going very well for me at school.
	I am satisfied with school.
	Motivational outcomes
Interest	
	It is important for me to do well in [mathematics/biology/chemistry/physics].
	I enjoy learning mathematics.
	I am interested in mathematics.
	Mathematics is one of my favorite activities.
Mathematics anxiety	
	When I run into difficulties during mathematics tests or examinations, I quickly lose confidence.
	I worry about getting poor grades in mathematics.
	Even when I am well prepared, I am very anxious before mathematics tests or examinations.
	I become tense when studying for mathematics tests or examinations.
	I become nervous when I cannot solve a mathematics problem.
	Academic climate variables
Boredom in mathematics class	
	In mathematics lessons, I am very bored.
	In mathematics lessons, I often think of things not related to my mathematics lesson.
	Mathematics homework is so boring that I do not want to do them.

Variable	Wording
Disciplinary climate	
	In our mathematics class, students interrupt the teacher.
	In our mathematics class, students talk loudly.
	In our mathematics class, students horse around.
Academic standards	
	Our mathematics teacher often asks questions that cannot be answered spontaneously but require us to think deeply.
	Our mathematics teacher gives us problems that do not have a single correct solution and asks us to explain our reasoning.
	Our mathematics teacher gives us tasks that require time to think about the solution.
	Our mathematics teacher presents tasks in different ways so that we can see whether we have really understood the underlying mathematical idea.
	Our mathematics teacher varies tasks so that we can see what we have understood.
	Our mathematics teacher often gives us tasks in which finding the right approach is more important than simply doing the calculations.
	When practising in mathematics, we often apply what we have learned to different situations or contexts.
	Practice exercises in mathematics often show whether we have really understood the material.
Gender stereotypes	
	Who is better in mathematics?
	Who enjoys mathematics more?
	Mathematics is more important for a future career for…
Teachers attitudes and values	
Aims of schooling	
	Teachers should make great efforts to promote students' social competencies.
	A good teacher prioritizes the development of students' enjoyment of learning and their social integration.
Gender stereotypes	
	Who is better in mathematics?
	Who enjoys mathematics more?
	Who spends more time engaging with mathematics?
	Who is more talented in mathematics?
	Mathematics is rather a subject for…
	Mathematics is more important for a future career for…

Note: Own translation. For original version, see official scale documentation (28).

Table A5. Correlations between individual items and nomination frequencies (individual level)

	Friendship		Break-Time		Help		Rejection	
Items	in	out	in	out	in	out	in	out
				Subjective social integration				
Other students seem to like me.	0.27	0.28	0.21	0.19	0.18	0.12	-0.25	-0.13
I have many friends in my class.	0.36	0.42	0.32	0.29	0.2	0.16	-0.25	-0.1
I spend my breaks with other students.	0.23	0.24	0.25	0.23	0.2	0.14	-0.21	-0.1
I am often teased by other students. (rec.)	0.07	0.05	0.04	0.03	0.09	0.03	-0.16	-0.11
I like most students in my class.	0.26	0.29	0.22	0.22	0.21	0.16	-0.27	-0.22
Others enjoy spending time with me.	0.31	0.32	0.28	0.25	0.21	0.15	-0.26	-0.13
I feel lonely during breaks. (rec.)	0.17	0.17	0.15	0.12	0.13	0.08	-0.16	-0.07
				Social well-being in school				
I often feel like an outsider at school. (rec.)	0.23	0.22	0.19	0.14	0.15	0.09	-0.21	-0.09
In this school, it is easy to make friends.	0.22	0.27	0.16	0.16	0.12	0.12	-0.14	-0.07
I feel like I belong at this school.	0.18	0.2	0.15	0.15	0.16	0.12	-0.14	-0.1
I often feel uncomfortable and out of place at this school. (rec.)	0.16	0.16	0.15	0.12	0.17	0.09	-0.16	-0.1
I am liked by other students in this school.	0.21	0.24	0.18	0.17	0.16	0.12	-0.19	-0.08
I feel lonely at this school. (rec.)	0.18	0.17	0.16	0.12	0.15	0.09	-0.17	-0.09
I feel happy at this school.	0.16	0.19	0.15	0.15	0.17	0.12	-0.14	-0.12
Things are going very well for me at school.	0.13	0.14	0.11	0.12	0.19	0.09	-0.14	-0.11
I am satisfied with school.	0.1	0.13	0.11	0.11	0.15	0.09	-0.12	-0.12

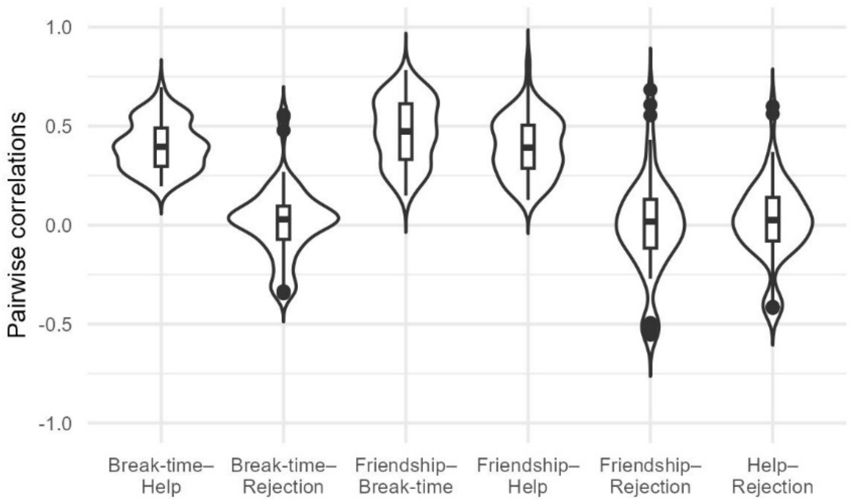

Figure A1. Distribution of pairwise correlations between network features of the different network types

Supplement B (Network features list)

(see separate .xlsx file)

Supplement C (Clustering procedure)

We applied a k-medoids clustering algorithm to the 314 x 314 correlation matrix between all pairs of network indicators, separately for each of the four networks (friendship, break-time, help, and rejection). To increase confidence in the cluster solution and make it comparable between networks, we tried to find a clustering solution that would be relatively consistent across the four networks. To this end, we first assessed the total within-cluster dissimilarity and silhouette width (1) of cluster solutions with k = 2 to k = 10 clusters. Silhouette width (1) is the proportion between the degree to which a feature is more similar to the same cluster than to the next closest cluster and the dissimilarity to all features of the next closest cluster. A higher silhouette width means that the features are clearly clustered, while a low silhouette width means that the solution does not reveal clear clusters. Furthermore, within-cluster dissimilarity is the distance between all features within a cluster and generally decreases as clusters become smaller. Thus, with an increasing number of clusters, this quantity decreases. An obvious elbow in the plot of total within-cluster dissimilarities across cluster solutions denotes a situation in which adding another cluster does not improve the clustering much. We inspected elbow plots of within-cluster dissimilarity (using a method proposed in ref. 2) and silhouette scores as well as the allocation of features to the clusters. We searched for a clustering solution that would be acceptable for all of the networks. Thus, we compared the allocation of network features to the different clusters for various cluster solutions. On the basis of these comparisons, we decided for six clusters, as the cluster composition seemed to be most comparable between the four networks. Our final decisions about how to allocate the network features to each of the six clusters were based on how they were allocated in the majority of the networks.

1. Rosseeuw, P. J. Silhouettes: A graphical aid to the interpretation and validation of cluster analysis. *J. Comput. Appl. Math.* **20,** 53–65 (1987).
2. Kolesnichenko, P. V., Zhang, Q., Zheng, C., Fuhrer, M. S. & Davis, J. A. Multidimensional analysis of excitonic spectra of monolayers of tungsten disulphide: toward computer-aided identification of structural and environmental perturbations of 2D materials. *Mach. Learn.: Sci. Technol.* **2,** 25021; 10.1088/2632-2153/abd87c (2021).

Supplement D (Estimation procedure of the elastic net regression models)

Model training and hyperparameter tuning were conducted in R using the caret package89. The data were split into training (80%) and testing (20%) data uniformly at random. By performing out-of-sample validation of our results on the hold-out data, we effectively controlled for overfitting. Hyperparameter tuning was conducted in the training data using tenfold cross-validation. We performed a grid search across a range of values for alpha from 0.1 to 1 with increments of 0.1 and for lambda with a sequence of nine values optimized by the “glmnet” algorithm specifically for each regression model. The best hyperparameters for the final model were chosen on the basis of the lowest root mean square error (RMSE) from cross-validation. Table G3 presents the penalty parameters for all optimized models. For each of the outcome variables, on the basis of the complete training data and the optimized hyperparameters, we finally fit a) a model with only the control variables (*Control variables only model*), b) a model including the control variables, friendship density, and centralization as “gold standard” indicators (*Gold standard model*), c) a model including the control variables and all network features (*All network features model*), d) a model including the control variables excluding school type and all network features (*Track excluded model*), and e) a model similar to c), additionally including all interactions between network features and school track (using a dummy for the highest track and a dummy for the lowest track, *Interactions model*) because school tracks in Germany are often deemed profoundly different developmental contexts.

Table D1: Final tuning parameters for elastic net regression of network features and control variables

	alpha	lambda
**Socio-emotional variables**		
Social Integration (M)	0.1	0.176
Social Well-being (M)	0.1	0.334
Teacher Stress (M)	1.0	0.067
**Academic climate**		
Discipline Mathematics (M)	0.3	0.189
High standards (M)	0.6	0.069
Boredom Mathematics (M)	0.5	0.085
Gender stereotypes students	0.3	0.113
**Academic achievement level and heterogeneity**		
Mathematics (M)	0.5	0.055
Physics (M)	0.4	0.052
Chemistry (M)	0.4	0.052
Biology (M)	0.6	0.053
Mathematics (SD)	0.5	0.109
Physics (SD)	0.5	0.100
Chemistry (SD)	0.9	0.050
Biology (SD)	0.8	0.062
**Motivation**		
Interest Biology (M)	0.6	0.059
Interest Chemistry (M)	0.8	0.096
Interest Physics (M)	1	0.055
Interest Mathematics (M)	0.2	0.125
Mathematics Anxiety (M)	0.2	0.136
**Instruction and Teacher Variables**		
Small group instruction	0.8	0.083
Individual study	1	0.042
Whole classroom instruction	0.4	0.116
Interdisciplinary learning	0.5	0.104
Free study time	0.7	0.087
Peer tutoring	0.9	0.032
Weekly plan	0.5	0.106
Project-based learning	0.6	0.096
Heterogeneous grouping	0.5	0.112
Homogeneous grouping	0.3	0.131
Gender stereotypes teacher	0.8	0.065
Importance fostering social competences	0.4	0.101
Fostering joy of learning and social integration	0.6	0.095

Supplement E (Explanatory power for all outcomes)

Table E1. Predictive power of social network models compared with the control variables only models for all 33 outcomes

Outcome	All network features model (track included)					Track excluded model			
	ΔRMSE	*p*	Regression R^2	Prediction R^2	ΔR^2	ΔRMSE	*p*	Prediction R^2	ΔR^2
Socio-emotional variables									
Social Integration (M)	**-0.107**	**<.001**	0.432	0.380	0.176	-0.115	<.001	0.378	0.192
Social Well-being (M)	**-0.062**	**0.002**	0.282	0.240	0.110	-0.061	0.002	0.240	0.109
Academic climate									
Boredom Mathematics (M)	-0.012	0.354	0.068	0.060	0.021	-0.012	0.354	0.060	0.021
Discipline Mathematics (M)	-0.020	0.066	0.056	0.087	0.048	-0.021	0.062	0.088	0.047
Gender stereotypes students	-0.012	0.444	0.140	0.088	0.025	-0.011	0.504	0.085	0.027
High standards (M)	0.002	0.604	0.196	0.211	-0.002	0.005	0.354	0.143	-0.001
Academic achievement level and heterogeneity									
Chemistry (M)	-0.018	0.016	0.686	0.704	0.018	-0.030	0.034	0.594	0.040
Mathematics (M)	-0.020	0.006	0.791	0.749	0.020	-0.045	<.001	0.672	0.054
Biology (M)	-0.022	0.018	0.710	0.601	0.006	-0.031	0.008	0.540	0.033
Physics (M)	-0.022	0.014	0.689	0.688	0.020	-0.035	0.026	0.610	0.043
Mathematics (SD)	0.001	0.770	0.031	0.049	0.007	0.001	0.794	0.049	0.007
Physics (SD)	0.001	0.832	0.032	0.002	-0.002	0.000	0.966	0.002	-0.001
Biology (SD)	0.004	0.504	0.060	0.000	-0.001	0.004	0.608	0.000	-0.001
Chemistry (SD)	-0.004	0.490	0.038	0.001	0.000	-0.004	0.446	0.000	0.000
Motivation									
Interest Biology (M)	-0.006	0.428	0.158	0.124	0.011	-0.014	0.198	0.090	0.025

Outcome	All network features model (track included)					Track excluded model			
	ΔRMSE	*p*	Regression R^2	Prediction R^2	ΔR^2	ΔRMSE	*p*	Prediction R^2	ΔR^2
Interest Mathematics (M)	0.016	0.142	0.036	0.014	-0.023	0.016	0.134	0.013	-0.025
Interest Chemistry (M)	-0.003	0.462	0.012	0.010	0.009	-0.004	0.334	0.010	0.009
Interest Physics (M)	-0.003	0.714	0.034	0.014	0.000	-0.004	0.588	0.014	0.000
Mathematics Anxiety (M)	-0.001	0.894	0.175	0.149	0.001	-0.001	0.900	0.149	0.000

Table E1. (contd.)

	All network features model (track included)					Track excluded model			
			Regression	Prediction				Prediction	
Outcome	ΔRMSE	*p*	R^2	R^2	ΔR^2	ΔRMSE	*p*	R^2	ΔR^2
			Instruction and Teacher Variables						
Homogenous grouping	-0.005	0.510	0.029	0.005	0.002	-0.002	0.840	0.006	-0.001
Heterogenous grouping	0.001	0.884	0.017	0.004	-0.003	0.002	0.708	0.004	-0.004
Peer tutoring	0.015	0.246	0.055	0.000	-0.002	0.003	0.826	0.000	-0.001
Whole classroom instruction	0.011	0.178	0.040	0.008	-0.018	0.005	0.440	0.005	-0.007
Free study time	-0.007	0.368	0.057	0.050	0.013	-0.001	0.814	0.034	0.012
Individual study	-0.004	0.482	0.022	0.018	0.007	-0.002	0.644	0.017	0.007
Weekly plan	0.014	0.310	0.119	0.077	-0.023	0.017	0.222	0.045	-0.026
Interdisciplinary learning	0.002	0.728	0.017	0.043	0.004	0.004	0.538	0.045	0.001
Project-based learning	0.000	0.902	0.020	0.012	0.001	-0.001	0.870	0.012	0.002
Small group instruction	0.010	0.220	0.065	0.025	-0.019	0.013	0.024	0.025	-0.023
Importance fostering social competences	0.006	0.180	0.020	0.003	-0.010	0.007	0.160	0.002	-0.007
Fostering joy of learning and social integration	0.002	0.490	0.012	0.027	0.005	0.003	0.298	0.022	0.004
Teacher Stress (M)	0.000	0.780	0.008	0.001	0.001	-0.001	0.500	0.001	0.000
Gender stereotypes teacher	0.004	0.560	0.049	0.007	-0.007	0.004	0.634	0.003	-0.007

Note. For both models, the explained variance R^2 in the test data (Prediction R^2) is given in addition to improvement in the root mean square error (RMSE) of prediction and improvement in prediction R^2 in comparison with the *Control variables only model*. For the *All network features model*, the table additionally shows the regression R^2 in the training data. Significance testing was based on 1,000 bootstrap iterations. All tests were two-sided.

Table E2. Predictive power for interaction models including interactions between network indicators and school track

Variables	ΔRMSE	p	ΔR²	p
Socio-emotional variables				
Social Integration (M)	-0.004	0.700	0.010	0.302
Social Well-being (M)	0.005	0.154	-0.008	0.126
Academic climate				
Boredom Mathematics (M)	0.006	0.348	-0.011	0.392
Discipline Mathematics (M)	0.008	0.028	-0.021	0.036
Gender stereotypes students	0.001	0.870	-0.004	0.644
High standards (M)	-0.001	0.694	0.008	0.290
Academic achievement level and heterogeneity				
Chemistry (M)	0.006	0.242	-0.007	0.256
Mathematics (M)	0.005	0.356	-0.006	0.348
Biology (M)	0.004	0.358	-0.001	0.934
Physics (M)	0.022	0.090	-0.027	0.094
Mathematics (SD)	-0.001	0.768	0.000	0.878
Physics (SD)	0.026	0.026	-0.040	0.022
Biology (SD)	-0.002	0.226	0.000	0.906
Chemistry (SD)	0.003	0.506	-0.002	0.802
Motivation				
Interest Biology (M)	0.013	0.146	-0.016	0.248
Interest Mathematics (M)	-0.008	0.382	0.009	0.390
Interest Chemistry (M)	-0.004	0.272	0.007	0.590
Interest Physics (M)	0.001	0.734	-0.002	0.660
Mathematics Anxiety (M)	0.006	0.134	-0.009	0.124
Instruction and Teacher Variables				
Homogeneous grouping	0.000	0.974	0.001	0.950
Heterogeneous grouping	-0.016	0.000	0.028	0.032
Peer tutoring	0.013	0.378	-0.009	0.552
Whole classroom instruction	0.005	0.484	0.005	0.598
Free study time	-0.005	0.142	0.006	0.228
Individual study	0.013	0.006	-0.018	0.166
Weekly plan	0.009	0.336	-0.019	0.292
Interdisciplinary learning	-0.018	0.064	0.000	0.890
Project-based learning	0.000	0.962	-0.001	0.928
Small group instruction	0.004	0.436	-0.003	0.772
Importance fostering social competences	-0.002	0.774	0.001	0.842
Fostering joy of learning and social integration	0.005	0.024	-0.009	0.200
Teacher Stress (M)	-0.001	0.610	0.000	0.942
Gender stereotypes teacher	-0.004	0.464	0.003	0.754

Note. Increase in predictive power compared to a model without interaction effects. **p* < .05. ***p* < .01. ****p* < .001.

Fig. E1: Aggregated feature importance for achievement outcomes

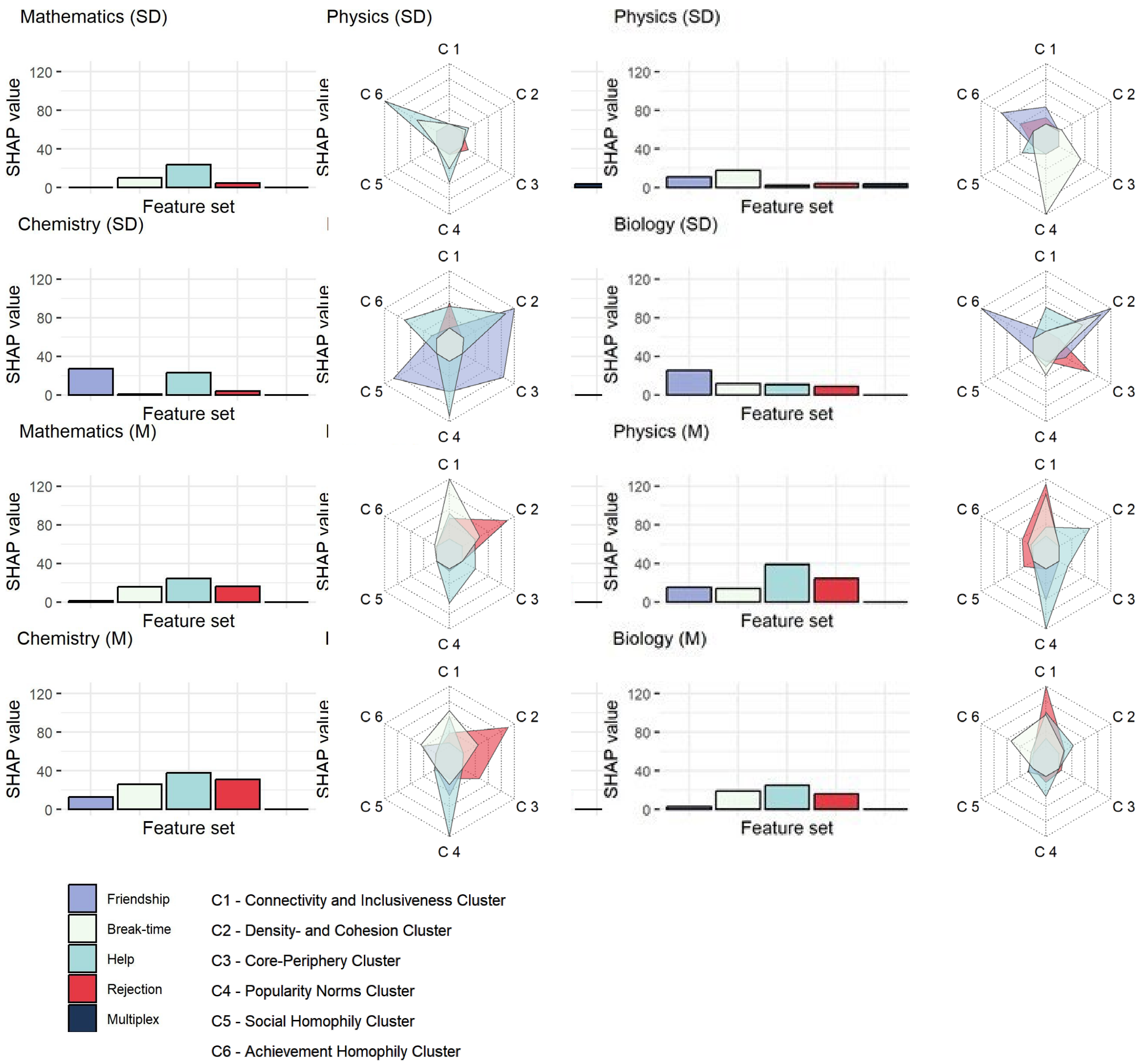

Fig. E2: Aggregated feature importance for emotional outcomes and teacher attitudes

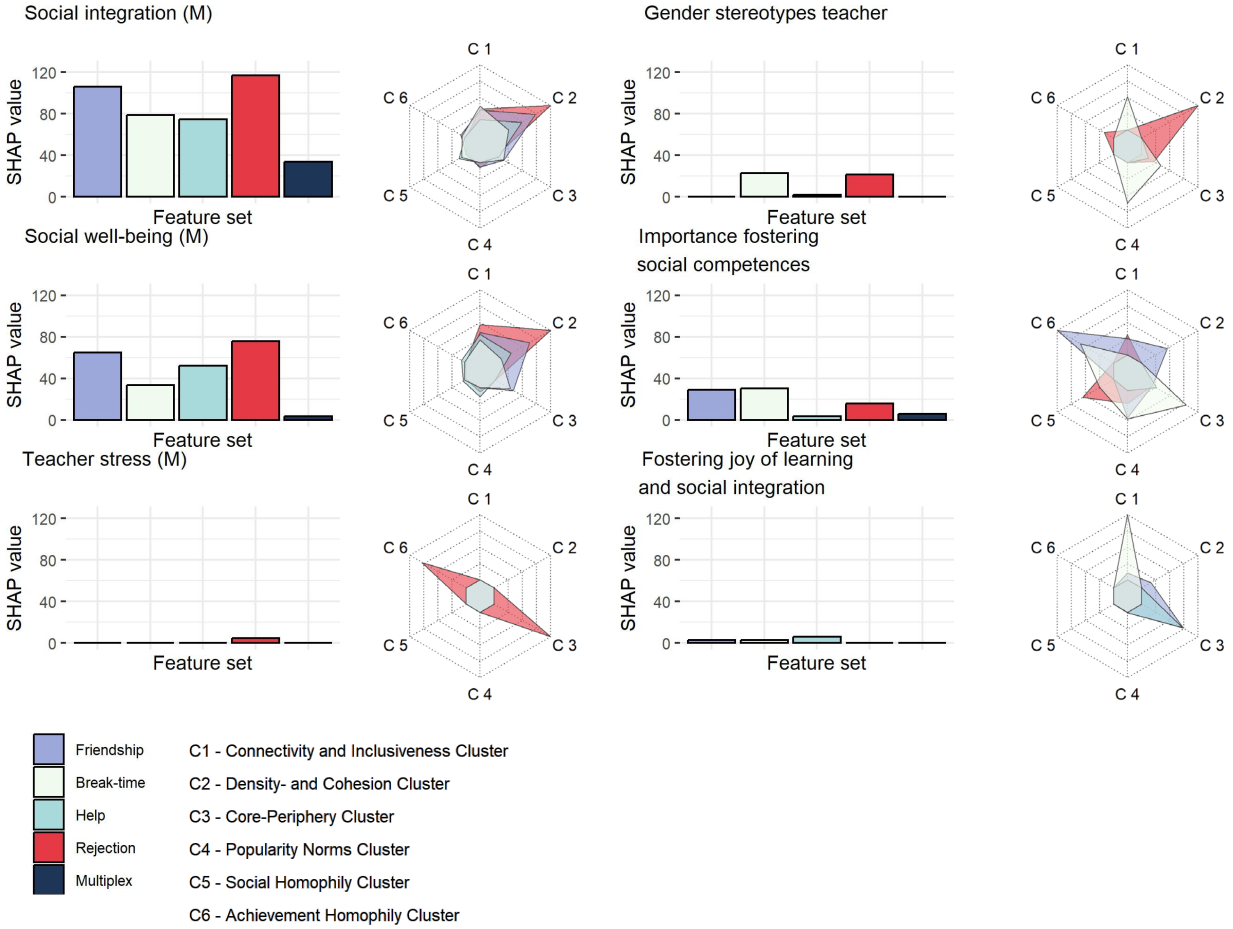

Fig. E3: Aggregated feature importance for motivational outcomes and disciplinary climate

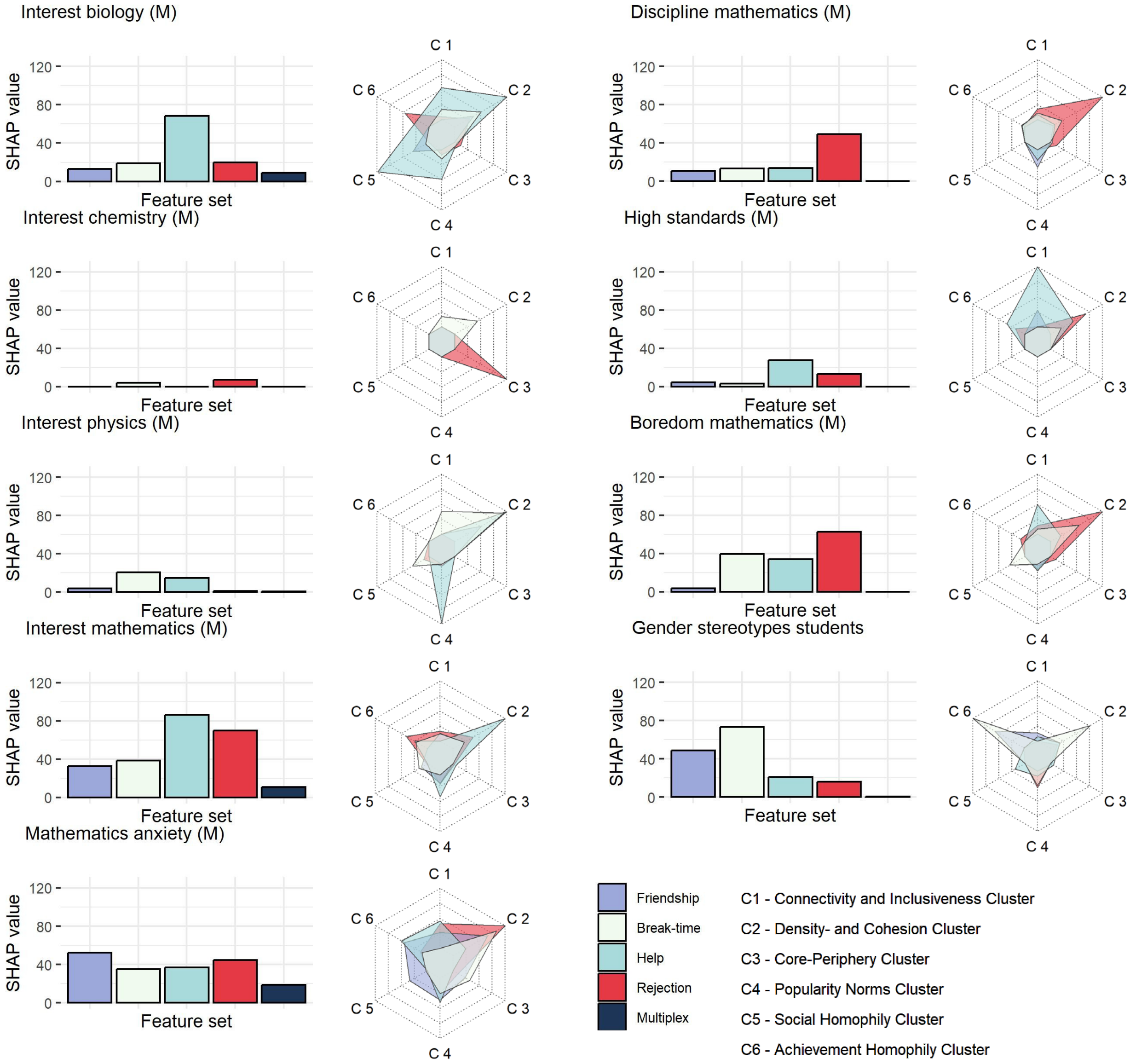

Fig. E4: Aggregated feature importance for instruction

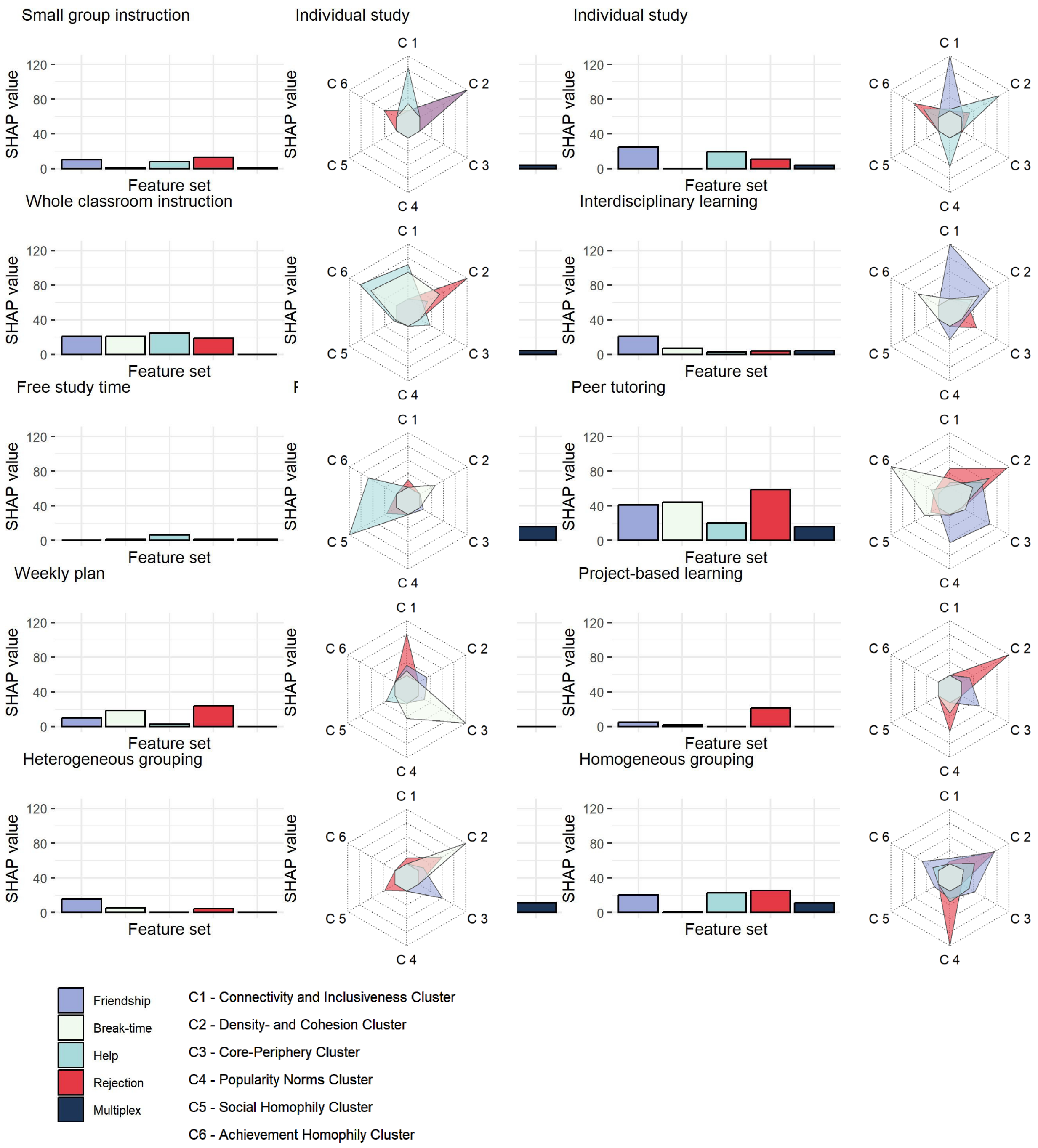

Supplement F (Individual Feature Importance)

We calculated the importance of individual network features. We did so by using Shapley additive explanations (SHAP) values[90]. For each network feature, Fig. F1 and F2 show the distribution of SHAP values across classrooms by means of a SHAP summary plot[91]. These plots show the features used to predict the outcome on the vertical axis (sorted by their cluster membership) and the local importance (SHAP value) of each feature on the horizontal axis. The dots represent one classroom each. The color of a dot corresponds to the feature value of the corresponding feature, including its sign. That is, a classroom could have a high value on a feature (red color in Figs. F1 and F2) and a negative SHAP value, meaning that this feature had a negative effect on the outcome. By contrast, if classrooms with high feature values had high SHAP values at the same time, the feature had an overall positive effect on the outcome. We found that some indicators showed large variance in the extent to which they drove the results of the estimation for different classrooms. Figures F3 and F4 aggregate the information by showing the absolute importance summed across all classrooms. Furthermore, the direction of the bars indicates the direction of the features' impact on an outcome.

Highlighting some consistent findings, these in-depth analyses corroborate findings from the above analysis of cluster importance. Achievement in physics, chemistry, and mathematics was higher in classrooms in which high-achievers in mathematics were more popular within the help network (note that the grading system in Germany is reversed: lower grades denote better performance). Thus, the mean achievement level in classrooms in which high achievers were asked for help more often was higher. Also, the inclusiveness of the help network was important: The higher the inclusiveness and the lower the proportion of students excluded from the network, the higher the achievement level.

Regarding the first cluster of the break-time network, it is apparent that a more inclusive network with a low disparity in nominations was related to higher achievement levels. Finally, the rejection network had also been related to the classrooms' achievement levels. The feature importance plots reveal:

Classrooms with many reciprocal dislikes (evidenced in both the Triad 3 counts and the reciprocity feature) have lower achievement levels. Also, a large dense group of students which are rejecting each other is related to lower achievement. In contrast, classrooms in which the rejections are less widely and less evenly distributed, fare better in terms of achievement levels.

Consistent patterns also emerged for socio-emotional outcomes. We found that the clustering coefficient (the density of ego-networks) of the friendship network played an important role in subjective social integration and well-being in school. Also, the proportion of empty triads, which is an indicator of sparseness, was negatively related to socio-emotional outcomes. Thus, high density and cohesion were positively associated with student social integration and well-being. On the contrary, the proportion of empty triads in the rejection network was positively related to both outcomes. Thus, a sparse network with lower density and cohesion in rejection nominations was beneficial for students.

Fig. F1: Individual feature importance for competence outcomes

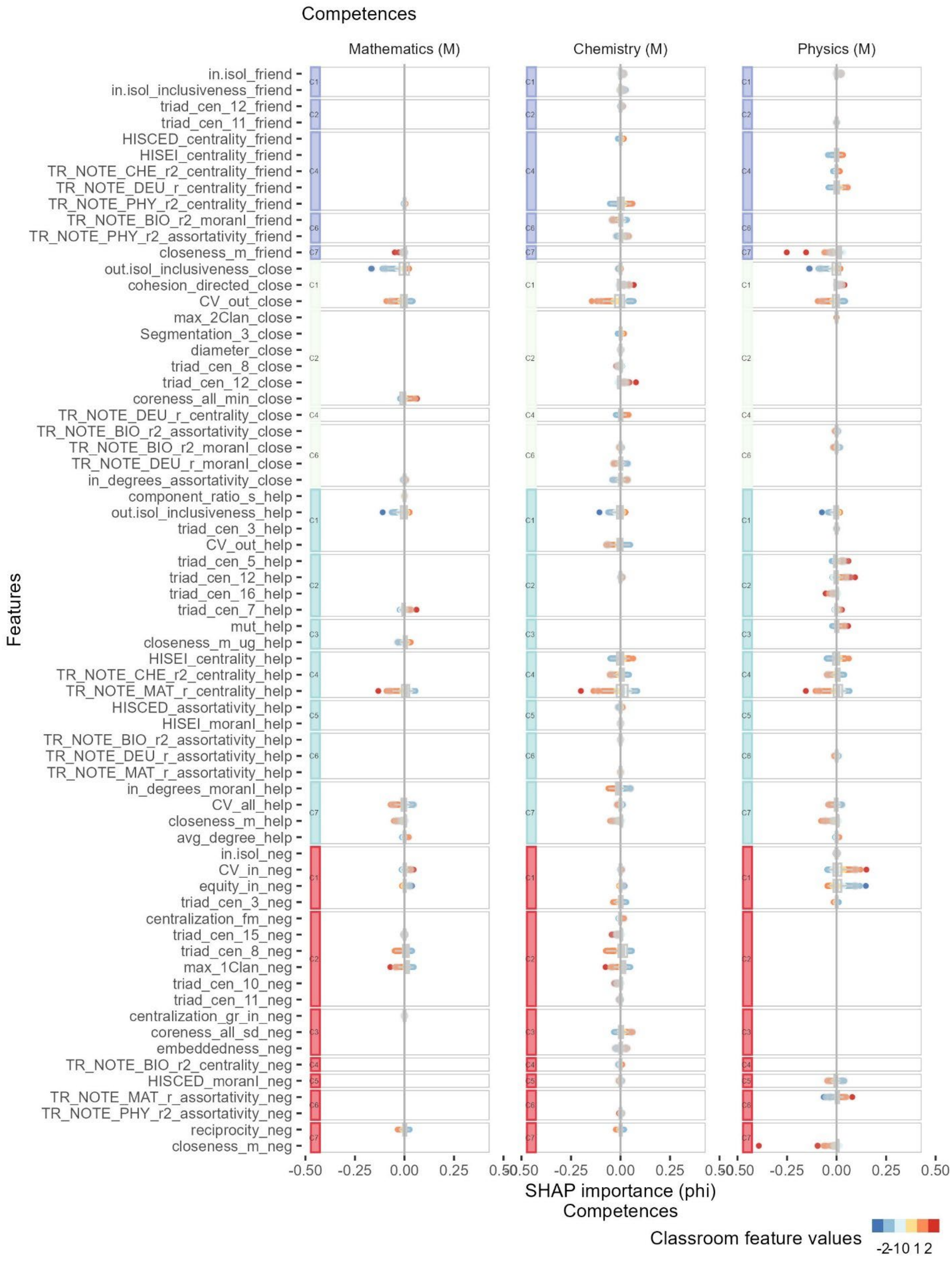

Fig. F2: Individual feature importance for socio-emotional outcomes

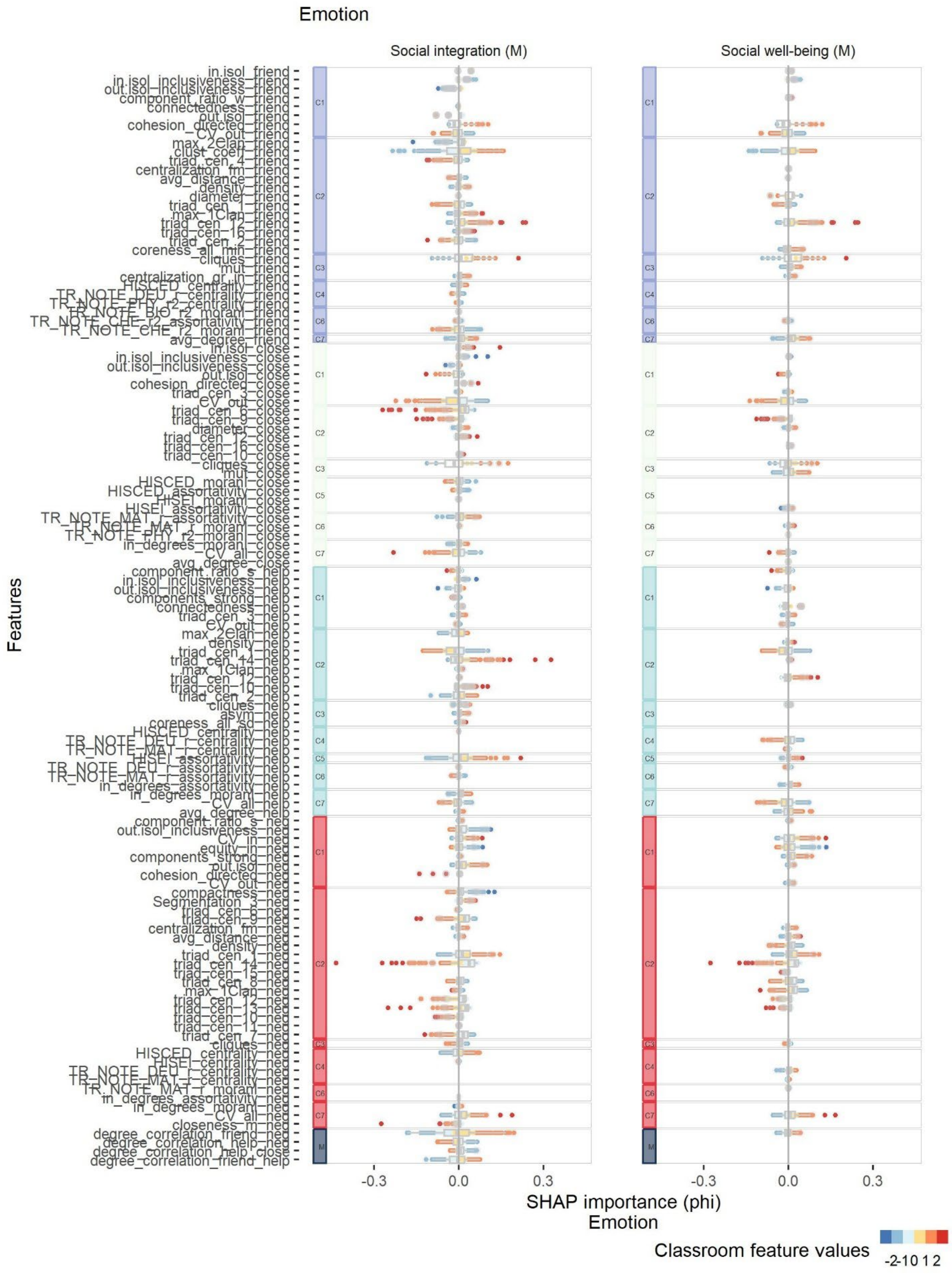

Fig. F3: Aggregated Feature Importance for all Clusters for Competence Outcomes

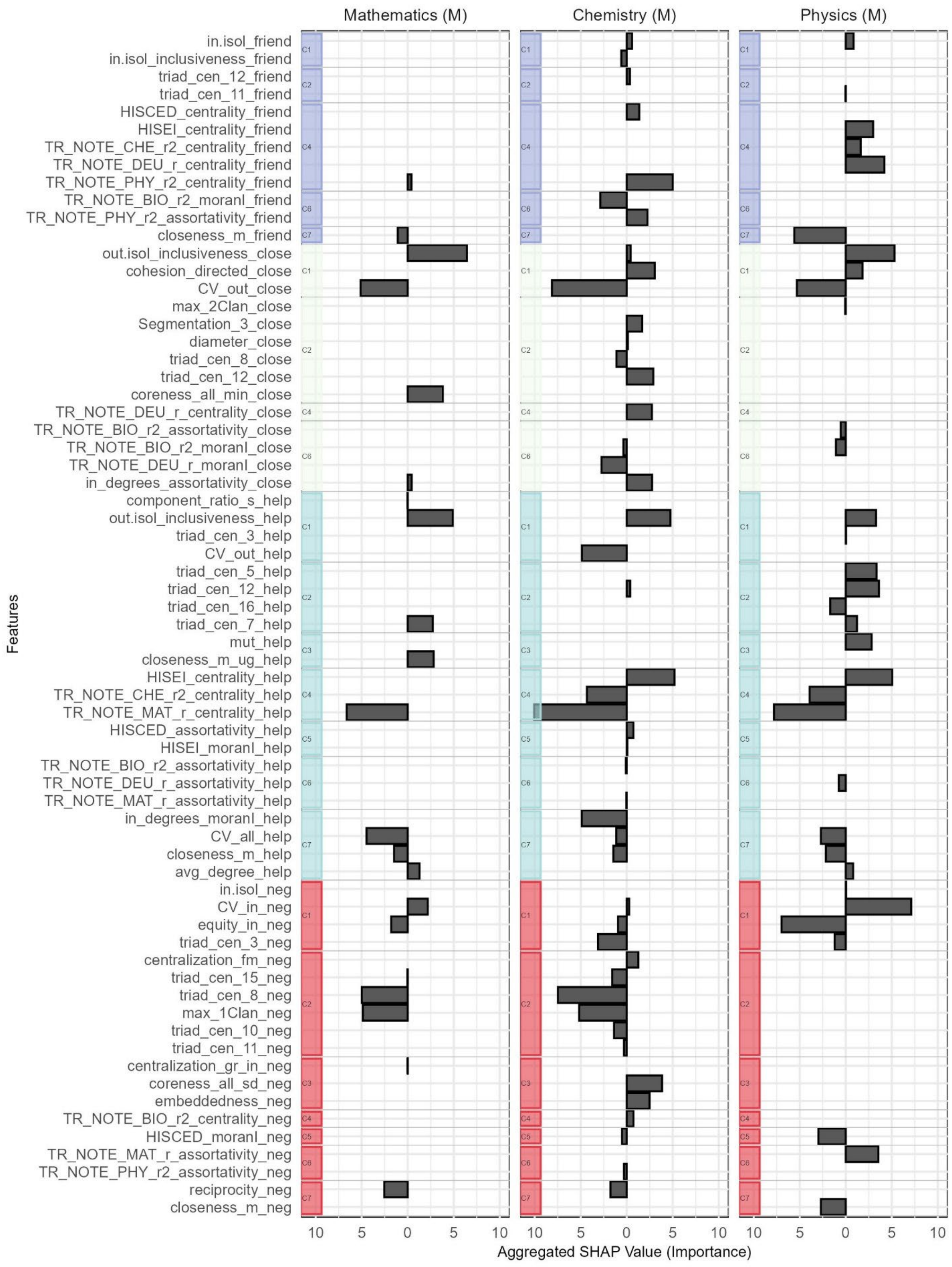

Fig. F4: Aggregated Feature Importance for all Clusters for Socio-Emotional Outcomes

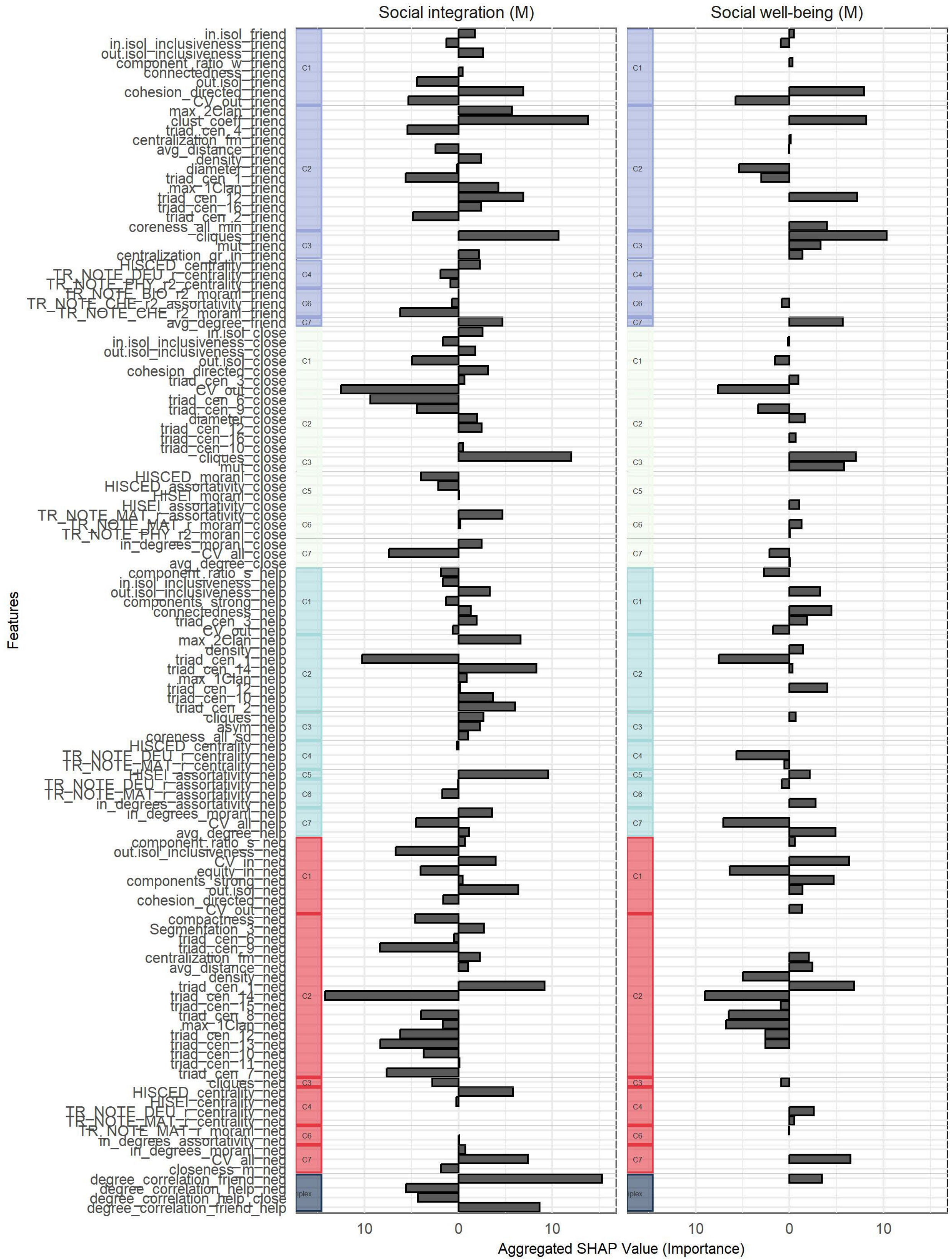

Supplement G (Robustness)

Tab. G1: Predictive power of network features beyond the control variables and the gold standard (25% cut-off for missing data)

Outcome variable	Gold standard analyses[a]				All network features[b]				Incremental predictive value[c]			
	ΔRMSE[d]		ΔR^{2d}		ΔRMSE[d]		ΔR^{2d}		ΔRMSE		ΔR^2	
Emotion												
Social Integration (M)	**0.043**	*	**8.9%**	***	**0.079**	**	**15.9%**	***	**0.036**	*	**7.0%**	***
Social well-being (M)	0.028		**5.5%**	*	**0.062**	**	**11.6%**	**	**0.034**	**	**6.1%**	***
Academic achievement												
Chemistry (M)	0.001		0.1%		**0.023**	**	**2.7%**	***	**0.023**	**	**2.6%**	***
Mathematics (M)	0.002		0.2%		**0.018**	**	**1.8%**	*	**0.016**	*	**1.6%**	***

Note. [a] Gold standard analyses include control variables and two network features: friendship density and friendship centralization. [b] All network features include control variables and all network features. [c] The incremental predictive value column compares the model with all network features with the model with gold standard features only. [d] Gold standard analyses and all network features were compared with a model with control variables only (not included in the table). Results were based on 372 classrooms in the hold-out data. Significance testing was performed using 1,000 bootstrap iterations. The reported significance levels indicate whether the bootstrapped 95%, 99%, or 99.9% confidence intervals excluded zero. All tests were two-sided.

*$p < .05$. **$p < .01$. ***$p < .001$.

Fig. G2: Prediction errors of the elastic net regressions for each outcome variable (25% cut-off for missing data)

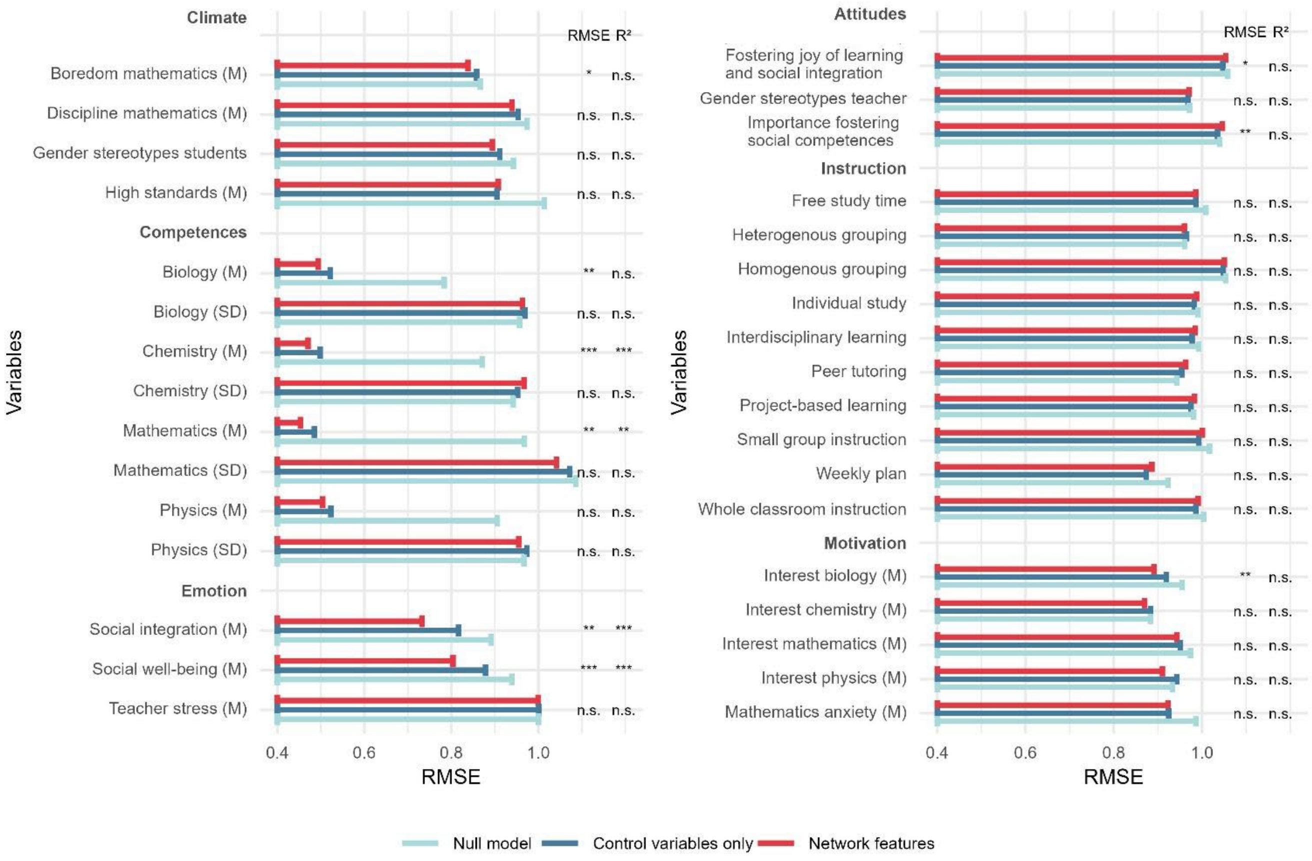

Note. The figure shows prediction errors (RMSE) across the three models with successively more variables (light-blue: null model, dark blue: model with control variables only, red: model including control variables and network variables). Stars denote p-values indicating whether there was a significant decrease in the prediction error of the model including network variables beyond the model with control variables only. For exact p-values, refer to Tab. E1 in Supplement E. Results were based on predictions for 372 classrooms in the hold-out data. Significance testing was based on 1,000 bootstrap iterations. All tests were two-sided.

**p* < .05. ***p* < .01. ****p* < .001.

Fig. G3 Aggregated feature importance by predictor set (25% cut-off for missing data)

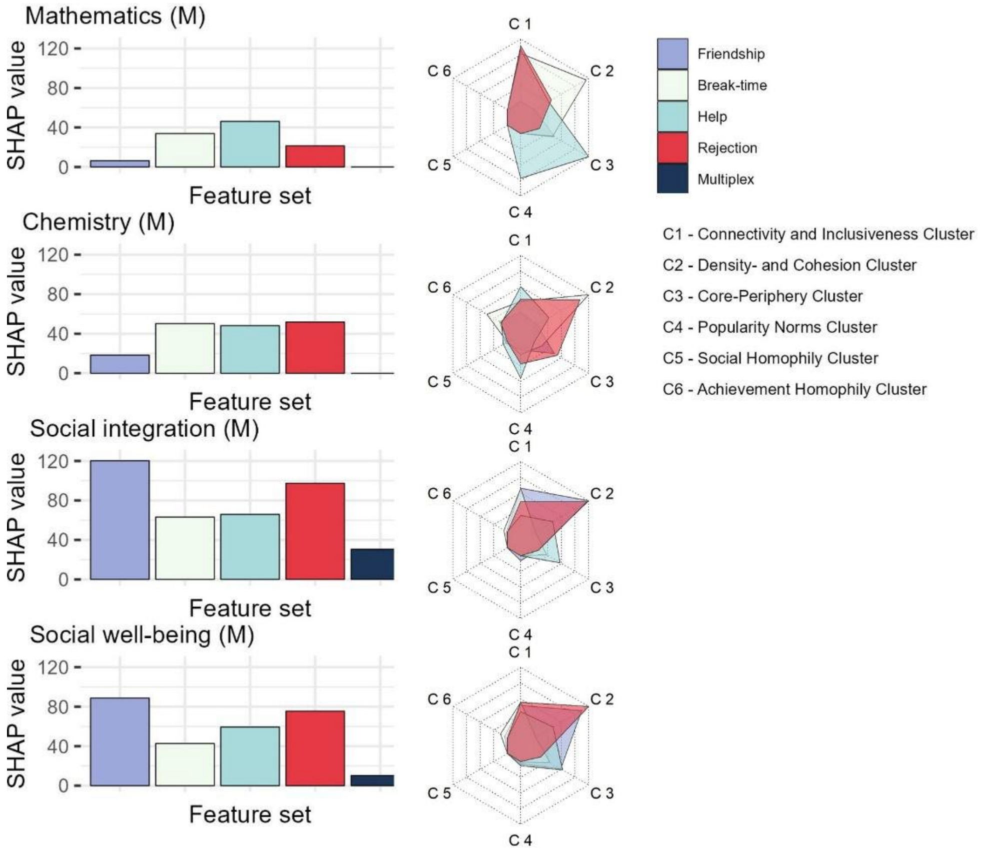

Note. Each row corresponds to one outcome. The total absolute SHAP value importance for all features within each of the networks (friendship, break-time, help, rejection, and multiplex) is displayed on the left side. The SHAP value importance for the empirical clusters (C1 – C6) within each of the four networks is depicted on the right side.
